# Assessing Odor Level when Using PrePex for HIV Prevention: A Prospective, Randomized, Open Label, Blinded Assessor Trial to Improve Uptake of Male Circumcision

**DOI:** 10.1371/journal.pone.0126664

**Published:** 2015-05-29

**Authors:** Vincent Mutabazi, Jean Paul Bitega, Leon Muyenzi Ngeruka, Corine Karema, Agnes Binagwaho

**Affiliations:** 1 Ministry of Health, Kigali, Rwanda; 2 Military Medical Insurance, Kigali, Rwanda; 3 Rwanda Military Hospital, Kigali, Rwanda; University of Pittsburgh, UNITED STATES

## Abstract

The PrePex is a WHO–prequalified medical device for adult male circumcision for HIV prevention. The Government of Rwanda was the first country to implement the PrePex device and acts as the leading center of excellence providing training and formal guidelines. As part of the Government’s efforts to improve PrePex implementation, it made efforts to improve the psychological acceptability of device by men, thus increasing uptake with VMMC in sub-Saharan Africa. Some men who underwent the PrePex procedure complained of foreskin odor while wearing the PrePex 3–7 days after it was placed. This complaint was identified as potential risk for uptake of the device. Researchers from Rwanda assumed there is a possible relation between the level of foreskin odor and patient foreskin hygiene technique. The Government of Rwanda decided to investigate those assumptions in a scientific way and conduct a trial to test different hygiene-cleaning methods in order to increase the acceptability of PrePex and mitigate the odor concern. The main objective of the trial was to compare odor levels between three arms, having identical personal hygiene but different foreskin hygiene techniques using either clear water with soap during a daily shower, soapy water using a syringe, or chlorhexidine using a syringe. One hundred and one subjects were enrolled to the trial and randomly allocated into three trial arms. Using chlorhexidine solution daily almost completely eliminated odor, and was statistically significant more effective that the other two arms. The trial results suggest that odor from the foreskin, while wearing the PrePex device, could be related to the growth of anaerobic bacteria, which can be prevented by a chlorhexidine cleaning method. This finding can be used to increase acceptability by men when considering PrePex as one of the leading methods for HIV prevention in VMMC programs.

**Trial Registration:**

Clinicaltrials.gov NCT02153658

## Introduction

Voluntary medical male circumcision (VMMC) is a one-time, low-cost intervention that has been shown to reduce men’s risk of HIV infection by 53%-60% and by up to 73% in post-trial observations [1–4]. Numerous studies on the topic have been published over the past two decades to elevate HIV prevention awareness, especially in sub-Saharan countries. The World Health Organization (WHO) and the Joint United Nations Program on HIV/AIDS (UNAIDS) have endorsed innovative approaches to VMMC uptake in settings in which HIV prevalence and incidence is high but male circumcision (MC) prevalence remains low in 14 target countries [5]. Recent modelling investigations commissioned by PEPFAR and UNAIDS have agreed on an action plan to reach 80% coverage of VMMC in 14 countries by 2015, which will entail performing roughly 20 million adult VMMCs by 2015, averting approximately 3.36 million new HIV infections and saving US$16.5 billion [6].

PrePex became the first medical device for adult male circumcision to receive WHO prequalification [7] as an alternative to conventional surgical circumcision methods already recognized by WHO. The PrePex VMMC procedure is bloodless and requires no injected anesthesia, suturing, or sterile setting in which controlled radial elastic pressure is applied to the foreskin between a rigid Inner Ring and an Elastic Ring to cut off distal blood flow. After 7 days, the necrotic foreskin and the device are removed.

The Government of Rwanda was the first country to implement the PrePex device and acts as the leading center of excellence providing training and formal guidelines. As part of the Government's efforts to improve the PrePex implementation, it made efforts to improve the psychological acceptability of the device by men to increase uptake with VMMC in sub-Saharan Africa.

PrePex researchers from Rwanda have assumed that there is a possible relation between levels of odor emanating from the foreskin to the foreskin hygiene technique. It was speculated that when a patient follows an appropriate foreskin hygiene technique while wearing the device, the odor before device removal (day 7) will be significantly lower than the odor of the foreskin of a subject who follows a less effective foreskin hygiene technique. Providing scientific evidence of the direct relation between odor and specific foreskin hygiene technique will allow VMMC–implementing bodies to create comprehensive and effective PrePex-related hygiene guidelines with concurrent potential reduction in complaints from men, thus increasing the acceptability rate.

## Materials and Methods

The protocol for this trial and supporting CONSORT checklist are available as supporting information: see S1 Protocol and S1 Checklist.

A randomized, controlled, blinded trial to assess the effectiveness of 3 different foreskin hygiene techniques was conducted at the Rwanda Military Hospital Kigali, Rwanda, from November 18th to December 4th, 2013.

### Participants

The trial was approved by the Rwanda National Ethics Committee, followed ICH-GCP guidelines, and was registered at clinicaltrials.gov (NCT02153658); however, trial registration was not completed prior to subject enrolment as the principal investigator was not aware for the need to register this type of trial.

Subjects were recruited to the trial after they were screened according to the following inclusion criteria:

- Age 21 to 49 years
- Subject wants to be circumcised
- Subject is uncircumcised
- Subject is able to understand the trial procedures and requirements
- Subject agrees to participate in one of three arms and to follow hygiene instructions
- Subject agrees to have independent, blinded smell reviewers in the same room on the removal visit
- Subject agrees that his partner will be interviewed via telephone
- Subject agrees to abstain sexual intercourse for 6 weeks post device removal
- Subject agrees to abstain from masturbation for 2 weeks post device removal
- Subject agrees to return to the health care facility for follow-up visits (or as instructed) after his circumcision for a period of 1 week
- Subject is able to comprehend and freely give informed consent for participation in this trial and is considered by the investigator to have good compliance for the trial
- Subject agrees to anonymous video and photographs of the procedure and follow up visits.

During screening, subjects were also subject to the following exclusion criteria:

- Active genital infection, anatomic abnormality or other condition, which in the opinion of the investigator prevents the subject from undergoing a circumcision
- Subject with the following diseases/conditions: phimosis, paraphimosis, warts under the prepuce, torn or tight frenulum, narrow prepuce, hypospadias, or epispadias
- Known bleeding/coagulation abnormality or uncontrolled diabetes as determined by questionnaire
- Subject who has an abnormal penile anatomy or any penile diseases
- Subject that in the opinion of the investigator is not a good candidate
- Subject does not agree to anonymous video and photographs of the procedure and follow up visits.

### Interventions

Subjects were randomly allocated to one of three different foreskin hygiene techniques (ratio 1:1:1): (1) control arm: subjects instructed to follow the current hygiene instructions of standard washing with soap and water during a daily shower; (2) soap arm: subjects instructed to clean the foreskin with soapy water once a day using a syringe; and (3) chlorhexidine arm: subjects instructed to clean the foreskin with diluted chlorhexidine (1%) once a day using a syringe.

The trial staff included 2 physicians, 4 procedure nurses, 3 smelling nurses, and 2 coordinators.

### Outcomes

The primary objectives of the trial were safety (total adverse events rate per foreskin cleaning technique in each trial arm) and efficacy (odor levels of three trial arms with identical personal hygiene but different foreskin hygiene techniques). Secondary objectives included subject perception of odor in each trial arm and provider perception of odor in each trial arm.

**Fig 1:**
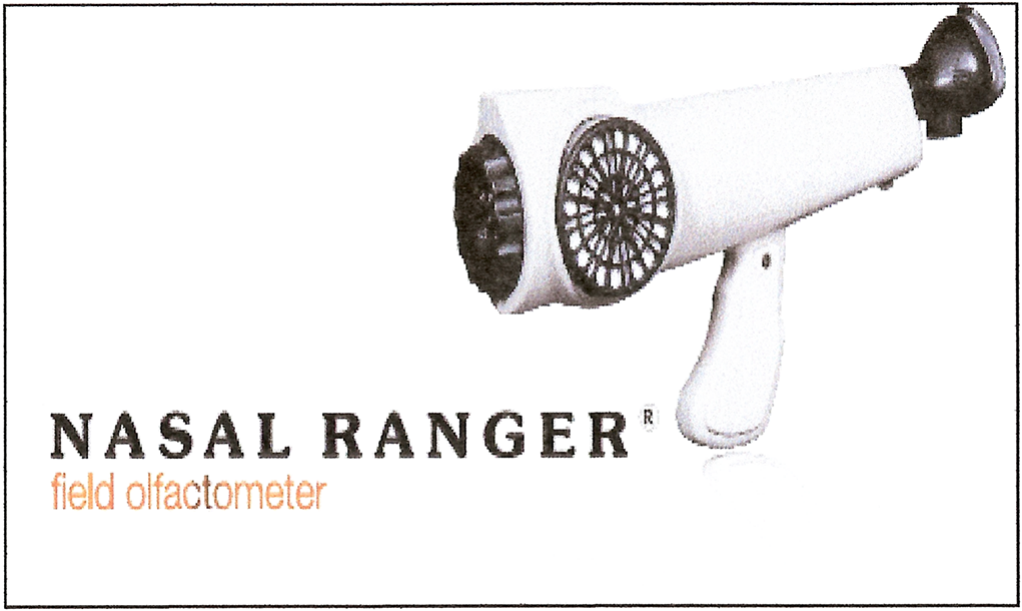
The Nasal Ranger device.

An odor visual analog scale (VAS) was used in order to quantify the odor levels sensed by the inspectors using the Nasal Ranger device (Fig 1). It ranged between 0 (no odor) and 5 (the highest odor level).

### Detailed procedures

All subjects’ base foreskin odor level were evaluated by three smellers using the Nasal Ranger device (St. Croix Sensory, Inc., MN, United States) [8], which allows objective quantification of odor levels using the dilution-to-threshold method [9]. The device measures and quantifies odor levels in the ambient air and works by enabling different levels of filtered air to flow into the device using scale dilution of 2, 4, 7, 15, 30, and 60 in which 60 is the most filtered state and one can smell odor only if it is very strong. The odor level was determined and documented by the filter level in which odor was first noticed by the smeller. The device was used by changing filters from the highest filter to the lower until first detection of odor.

A PrePex procedure was then performed by a qualified PrePex nurse, placing the device according to a standard PrePex procedure [10].

After PrePex placement, each subject attended a discharge session in which instructions were given on how to handle the device during the following 7 days, in addition to specific hygiene and cleaning instructions and a specific request not to share with others the hygiene technique communicated to him. To avoid disturbing the anesthetic 5% lidocaine cream, which is used during PrePex procedure, this session took place at least 30 minutes after the placement procedure, assuring the lidocaine cream was absorbed.

Control arm subjects were instructed to keep daily foreskin hygiene and wash the penis and foreskin gently with soap and then rinse without disturbing the device during showers, followed by drying with a towel. It was emphasized they should not use any other tool or method when cleaning the foreskin. Subjects in the soap arm were given a kit comprising a 20 mL syringe with no needle and standard soap so they could mix the soap in a bowl with clean water. Subjects from the chlorhexidine arm were provided with seven bottles of 20 mL diluted chlorhexidine (a bottle for each day of wearing the PrePex device) and a 20 mL syringe (without needle). Subjects in both the soap and chlorhexidine arms were instructed by a trial nurse how to use the syringe with the soap-water or chlorhexidine solution and rinse the inner part of the foreskin on a daily basis prior to or during their daily shower. Similarly to ear irrigation, subjects of both arms rinsed the given liquid under high pressure and shook the foreskin up and down while the liquid was inside. In order to remove excess residue, subjects were instructed to rinse the inner foreskin again with clean water only and dry with a towel. At the end of the instruction session each subject was then asked to demonstrate to the nurse how they would clean the foreskin by themselves.

Subjects arrived for follow-up visits on days 3, 5, and 7, in which three blinded ‘smellers’ used a Nasal Ranger. Odor was also evaluated by the PrePex operator before foreskin removal using the same odor scale, performing the odor assessment from a measured distance of 3–5 cm from the penis. Two rooms were designated for the follow-up visits, with one bed in each room. The subjects were queued according to their trial serial number (not specific for each arm), and on their turn, each subject entered the room and was instructed to pull off his pants under knee level, hold a big piece of fabric obstructing his torso and face, thus minimizing the probability that a blinded smeller could recognize subject’s characteristics. A separate coordinator was responsible to prepare the subjects in the room, documenting their trial number and the blinded smellers' odor result in a designated table form. Each blinded smeller smelled the subject's foreskin, according to predefined guidelines once, and then moved to the second room for the next subject smelling evaluation. The smellers went into the room one after the other in consecutive order, where they did not see the subject's face or the other smeller's results. Each smeller did not discuss their smelling results with the other smeller. The smelling procedure included using a clean gauze to grab the penis shaft and initially smelling without the Nasal Ranger device in order to get the sense of the odor to expect. The second smelling evaluation utilized the Nasal Ranger, starting to smell from the highest filtered air level to the lowest level. The Nasal Ranger was placed on the same distance from the foreskin for each smeller, 3–5 cm from tip of penis. The odor level was defined according to the Nasal Ranger filter number of which the smellers first notice a trace of odor. The discharge session occurred in the same manner as for day 0. Subjects returned on day 5 for another follow-up visit, which was conducted in the same manner as for the day 3 follow-up visit. The final trial visit was on device removal day, 7 days after placing the device. On this day the odor was expected to be the highest, therefore, in addition to the smelling evaluation by each smeller, a random sample of 29 subjects were blindly evaluated a second time, validating the repeatability of the odor evaluation by each smeller. The intra-tester reliability refers to the reliability for each smeller, and inter-tester reliability refers to the reliability between smellers. Questionnaires were used during visits 3, 5, and 7 to gather information on subjects' perspective of their odor and its effect on daily life. Additional odor assessment tests included questionnaires for subjects to quantify odor.

### Random sequence generation and allocation concealment

After signing the consent form, each subject was screened and randomized by the principal investigator into one of 3 trial arms described above. A randomization table was prepared beforehand using the RAND algorithm of excel. Allocation concealment was effected by using sealed envelopes that contained the allocation of intervention for each subject.

### Statistical analysis and sample size calculations

Although the trial design employed three different devices (with one smeller for each device), no replicates, and three arms, it is likely that the odor data will be non-normal. By collapsing the odor levels into 3 categories (low: < 3.9; medium: 4–6.9; high: > 7), a 3x3 matrix was constructed to estimate sample size by chi square. Thus, a sample size of 133 achieves 80% power to detect an effect size (W) of 0.3 using a 4 degrees of freedom chi square test with a significance level (alpha) of 0.05 (Pass 11, NCSS, LLC, Kaysville, UT, USA).

Data are presented via descriptive statistics. Binary data, such as adverse events success/fail criteria, are presented as a count and percentage together with an exact 95% confidence interval (CI). Adverse event rates were compared descriptively with the reported rates of similar treatments. Continuous data are represented by means or medians and standard deviations (SD). An alpha of 0.05 was used to ascertain statistical significance with all statistical tests two sided, and conducted using PASW 22 (IBM, Chicago, IL, USA). Fisher exact tests were used to compare odor levels between the three arms when comparing dichotomous parameters. Ordinal logistic regression was used to compare odor levels between the three arms when comparing ordinal parameters unless there were issues with goodness of fit and/or tests of parallel lines, in which case a Fisher’s exact test was performed. Bowker’s test of symmetry [11] was used to evaluate a random sample of subjects the second time on day 7 to validate if there was an agreement between smellers and within each smeller.

The primary analysis was performed on an intent-to-treat basis with additional per protocol analysis performed when patients were lost to follow-up or in cases of non-compliance.

## Results

**Fig 2:**
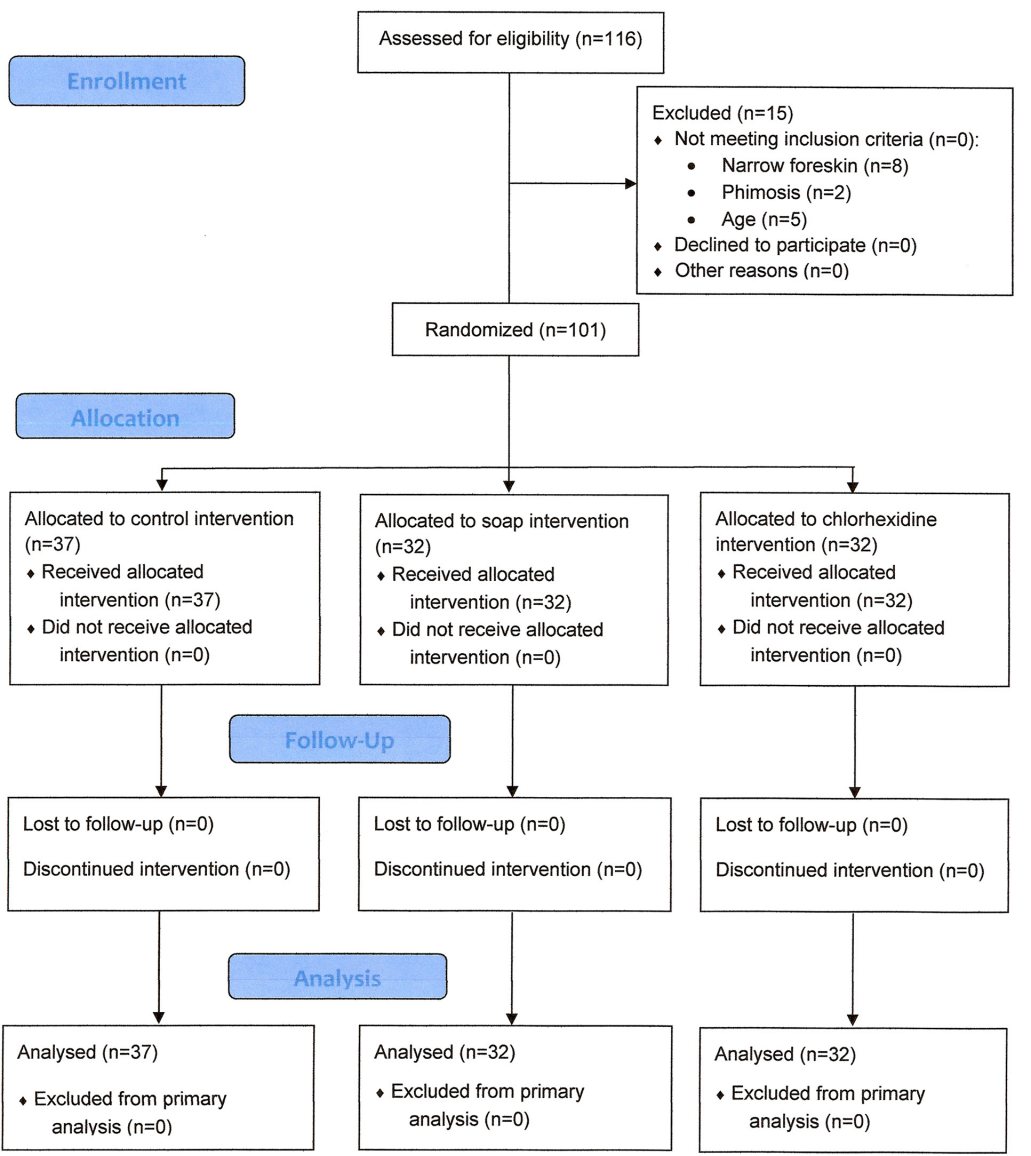
Flow of participants in the trial.

One hundred and one subjects were enrolled in the trial and randomly distributed between the 3 trial arms: 37 in control arm, and 32 each in the soap and chlorhexidine arms (Fig 2). Trial duration was 16 days, between November 18th through December 4th, 2013.

### Safety

There were no adverse events or complications during or after foreskin cleaning procedure for all subjects; therefore, all cleaning methods were found to be safe. There were no adverse events in the trial.

### Odor levels

Odor levels on day 7, measured by 3 blinded smellers, were significantly lower in the chlorhexidine arm compared to the other 2 arms (p = 0.033; Table 1), with the control arm showing the highest levels of odor (in the ordinal logistical regression there were failures with goodness of fit and parallel line testing).

**Table 1:** Odor levels for each arm determined by blinded smellers on day 7.

Nasal Levels	Odor VAS (Day 7)	Control	Soap	Chlorhexidine
< 3.9	Low	19 (51%)	15 (47%)	21 (66%)
4–6.9	Medium	7 (19%)	11 (34%)	10 (31%)
> 7	High	11 (30%)	6 (19%)	1 (3%)
Total (N)		37 (100%)	32 (100%)	32 (100%)

A random sample of subjects was evaluated a second time on day 7 to validate if there was an agreement between smellers and within each smeller. No statistically significant differences were found between the first and second rounds of smelling tests (p = 0.13), suggesting that the results for each smeller were reliable.

The odor scale as determined by the operators just before the removal procedure on day 7 with their nose without using the Nasal Ranger, was lowest in the chlorhexidine arm (Table 2). When odor categories 2–5 were collapsed into one category and the arms compared using ordinal logistic regression, the difference between the chlorhexidine and soap arms was significant (p = .008) but not statistically significant between the chlorhexidine and control arms (odds ratio for lower to higher odor levels for the soap arm versus the chlorhexidine arm: 0.25, 95% confidence intervals: 0.095–0.70). Goodness of fit results and parallel arm testing were not statistically significant (Nagekerke pseudo R^2^: 0.083).

**Table 2:** Odor levels for each arm determined by operators.

Odor VAS (Day 7)	Control	Soap	Chlorhexidine
0	18 (49%)	12 (38%)	22 (69%)
1	15 (40%)	13 (40%)	8 (25%)
2	3 (8%)	5 (16%)	2 (6%)
3	0 (0%)	2 (6%)	0 (0%)
4	1 (3%)	0 (0%)	0 (0%)
5	0 (0%)	0 (0%)	0 (0%)
Total	37 (100%)	32 (100%)	32 (100%)

Most of the operators (69%) did not smell any odor when examining the chlorhexidine arm and only 25% noticed minimal odor (VAS 1).

Subjects were asked questions during the follow up visits, the results of which are for day 7 shown in Table 3. While 50% and 30% of the subjects in the control and soap arms, respectively, experienced odor, only 7% in the chlorhexidine arm experienced odor; this was statistically significant (Question 1, Fisher exact test: p<0.0001).

**Table 3:** Subjects’ feedback during day 7 visit.

Question	Control	Soap	Chlorhexidine
1. "Have you experienced odor?"	Yes: 50% (18/36)	Yes: 30% (9/30)	Yes: 7% (2/31)
2. Has anyone else noticed the odor?	Yes: 8% (2/36)	Yes: 3% (1/30)	Yes: 0% (0/31)
3."Do you regret choosing PrePex?" and "Would you recommend the device to your friends or family?"	97%-100% answered no and yes, respectively.		

In regard to the remaining two questions, few participants reported that others had noticed the odor with the highest percentage in the control arm. Only 0–3% of participants regretted choosing the PrePex device but 97–100% would recommend the procedure to their friends regardless of the cleaning technique used nor the odor they may have sensed.

## Discussion

The trial results clearly demonstrate that there is a statistically significant reduction of odor when using chlorhexidine compared to intensive washing with soapy water or normal hygiene practice. It is suspected that during the PrePex procedure, necrotic tissue is developed as a result of an ischemic process, creating an intact, isolated internal gap—the sub-prepuce space (between the inner foreskin and the glans)—which provides an optimal environment for growth of anaerobic bacteria, which are known to produce unpleasant odor. This isolated space is breached once a wound is created by the device, creating an entry point and enabling bacteria to penetrated and thrive in it. The chlorhexidine cleaning method was found to be effective in preventing odor created along the entry point on the necrotic foreskin.

All cleaning methods were found to be safe. The results show that with appropriate foreskin technique, odor can be prevented. The results also showed that even in the two arms that used soap and water, the odor levels were not high enough for the majority of patients to regret selecting the PrePex circumcision method, with all reporting they would recommend the procedure to their friends.

The questionnaire results and operator odor scale determination support the nasal ranger findings that the foreskin cleaning technique, which includes the use of chlorhexidine, is superior to the other two methods; it is presumed that the chlorhexidine suppresses anaerobic growth and thus prevents odor.

We found that even men that had a higher level of odor did not find this level to be a reason to ask to stop the procedure. Therefore, we would make the following operative recommendations: Pending further trials using chlorhexidine, we recommend that men who undergo a PrePex circumcision be educated regarding foreskin hygiene technique using the diluted chlorhexidine approach. We will follow the feedback received from the field and adjust our actions accordingly.

The trial focused only on three specific foreskin hygiene techniques: one control and two new techniques. There is a need to investigate whether additional cleaning techniques or cleaning materials will have the same effect as those presented in the current trial. One example is using salt water for the daily care, a solution that can be simpler for patients as the ingredients are already available, as the use of chlorhexidine requires that patients be given material to take home. In addition, the Nasal Ranger device was not specifically designed for smelling localized odors but is used for detecting environmental odors. However, we believe that use of three independent smellers and the high agreement between their odor determination and the use of very controlled smelling conditions and instructions, demonstrated the validity of using Nasal Ranger for this use. Nevertheless, there could have been inconsistencies in odors over the trial period. Another trial limitation concerns the blinding method and the subject’s confidentiality when obscuring his face and torso. We cannot be completely assured that the smellers did not identify characteristics in the genital area when testing the smell in regard to the inter- and intra-rater tests.

## Conclusions

Providing scientific evidence of the relation between odor and foreskin hygiene will allow implementing bodies to create comprehensive and effective PrePex-related hygiene guidelines and reduce the potential of complaints from men, increasing acceptability of PrePex, and allowing to gain a larger uptake with VMMC programs when using PrePex in existing and future projects.

## Supporting Information

S1 Checklist CONSORT 2010 Checklist. (DOC)

S1 Protocol Study Protocol. (DOC)
