# Supplementary material for: Assessing Odor Level when Using PrePex for HIV Prevention: A Prospective, Randomized, Open Label, Blinded Assessor Trial to Improve Uptake of Male Circumcision
Source: PLoS One. 2015 May 29;10(5):e0126664. doi: 10.1371/journal.pone.0126664 (PMC4449041; doi:10.1371/journal.pone.0126664)
Supplement: S1 Protocol — (DOC) [file pone.0126664.s002.doc]

­­­­­­­­­­­­­­­­­­­­­­­­­­­­­­­­­­­­­­**Protocol Title**

Assessing Odor Level when Using PrePex for HIV Prevention: A Prospective, Randomized, Open Label, Blinded Assessor Trial to Improve Uptake of Male Circumcision

**Protocol Date**

August 2013

**Protocol Number**

RMC-10

**Protocol Version**

01

Study Sponsor: **Government of Rwanda** & Circ medtech limited

Principal Investigators:

Dr. Vincent Mutabazi [mutabazivincent@yahoo.com](mailto:mutabazivincent@yahoo.com) Tel 0788410827

Dr. Jean Paul Bitega [drbitega@yahoo.fr](mailto:drbitega@yahoo.fr) Tel 0783004100

Dr. Leon Muyenzi Ngeruka [dr_ngemleon@yahoo.com](mailto:dr_ngemleon@yahoo.com) Tel 0788501063

# This protocol represents intellectual property of the Government of Rwanda, Ministry of Health. Use of whole of parts of this protocol require approval from the Ministry of Health, and must be properly credited.

#

# Study Synopsis

The PrePex device was accepted for the World Health Organization (WHO) list of prequalified male circumcision (MC) devices and was listed on 31 May 2013.

PrePex became the first medical device for adult male circumcision to receive WHO prequalification as an alternative to the conventional surgical circumcision methods already recognized by WHO.

Voluntary Medical Male Circumcision (VMMC) is a one-time, low cost intervention that shown to reduce men’s risk of HIV infection by approximately 70 percent. The WHO recommends VMMC as part of a comprehensive package of HIV prevention services. The WHO prequalification of PrePex represents an unprecedented opportunity to further the safe and rapid scale up of voluntary medical male circumcision programs [Official statement by the Office of the U.S. Global AIDS Coordinator].

Up to date more than 7,500 MCs were performed using the PrePex device. Rwanda, Zimbabwe and Uganda have completed the WHO clinical roadmap, scientifically validating the safety, efficacy, acceptability and superiority (over dorsal slit, on both measures of time and safety) of PrePex, other countries such as Kenya, Botswana and Mozambique are currently conducting PrePex pilot studies.

A concern that was raised by WHO TAG consulting members regarding the potential acceptability of PrePex by men involved the potential of strong odor extraction from the necrotizing foreskin. PrePex researchers from Rwanda have assumed that there is a possible relation of the level of odor to the personal hygiene and foreskin washing technique. It is speculated that when a patient follows an appropriate washing technique of the foreskin while wearing the device, the odor before device removal (day 7) will be redundant and significantly lower than the odor of the foreskin of a patient that does not follow such washing instructions and technique. Providing scientific evidence of the direct relation between odor and personal hygiene will allow VMMC implementing bodies to create a comprehensive and effective PrePex personal hygiene protocol and increase acceptability of the VMMC.

This clinical study will include the following objectives:

- To compare the different odor levels when examining 3 arms with identical personal hygiene but different foreskin hygiene techniques
- To determine a detailed and validated foreskin cleaning procedure protocol, that will ensure low odor without compromising safety

To compare the different perception of the subject’s partner relating to the odor when examining 3 arms with identical personal hygiene but different foreskin hygiene techniques.

This protocol describes the endpoints and methods of assessing those objectives.

**Study Design and Title:**

Assessing Odor Level when Using PrePex for HIV Prevention: A Prospective, Randomized, Open Label, Blinded Assessor Trial to Improve Uptake of Male Circumcision.

**Planned Number of Subjects:**

At least one hundred (100) adult men scheduled to undergo VMMC.

**Study Duration:**

Study duration per subject will be up to 1 week (Placement visit and Removal visit after 7 days).

**Primary Objectives:**

- To compare the different odor levels when examine 3 arms of patients undergoing PrePex circumcision with same personal hygiene and with different foreskin hygiene techniques.

**Secondary Objectives**

- To determine an absolute odor level per each arm
- To determine a penis cleaning procedure guidelines safety and efficacy
- To compare the different perception of the subject’s partner relating to the odor when examine 3 arms of patients undergoing PrePex circumcision with same personal hygiene and with different foreskin hygiene techniques.

**Eligibility Criteria:**

**Inclusion Criteria:**

- Ages – 21 to 49 years
- Subject wants to be circumcised
- Uncircumcised
- Able to understand the study procedures and requirements
- Agrees to participate in either arm and to follow hygiene instructions
- Agrees to have Independent blinded smell reviewers in the same room on Removal visit
- Agrees that his partner will be interviewed via telephone
- Agrees to abstain sexual intercourse for 6 weeks post device removal
- Agrees to abstain from masturbation for 2 weeks post device removal
- Agrees to return to the health care facility for follow-up visits (or as instructed) after his circumcision for a period of 1 week
- Subject able to comprehend and freely give informed consent for participation in this study and is considered by the investigator to have good compliance for the study
- Subject agrees to anonymous video and photographs of the procedure and follow up visits.

**Exclusion Criteria:**

- Active genital infection, anatomic abnormality or other condition, which in the opinion of the investigator prevents the subject from undergoing a circumcision
- Subject with the following diseases/conditions: phimosis, paraphimosis, warts under the prepuce, torn or tight frenulum, narrow prepuce, hypospadias, epispadias
- Known bleeding / coagulation abnormality, uncontrolled diabetes, by questionnaire
- Subject who have an abnormal penile anatomy or any penile diseases
- Subject that to the opinion of the investigator is not a good candidate
- Subject does not agree to anonymous video and photographs of the procedure and follow up visits.

**Study Site:** The study will take place at Rwanda Military Hospital.

**Study arms:**

The subjects will be randomized into 3 study arms, using a randomization table and sealed envelopes:

**Arm 1:** A control arm – subjects instructed to follow the current hygiene instructions of standard washing with soap and water during a daily shower. They will get a soap to take home and will be questioned daily to make sure they followed the cleaning instructions.

They might be requested to wash themselves at the hospital before the actual Removal visit starts.

**Arm 2:** subjects instructed to clean the foreskin with soapy water once a day using a syringe.

They will get a soap and required accessories to take back home and will be questioned daily to make sure they followed the instructions.

Subjects may be requested to wash themselves at the hospital before the actual Removal visit starts.

**Arm 3:** subjects instructed to clean the foreskin with diluted chlorhexidine (1%) once a day using a syringe. They will get chlorhexidine in tubes and required accessories to take back home and will be questioned daily to make sure they followed the instructions.

Subjects may be requested to wash themselves at the hospital before the actual Removal visit starts.

**Independent Blinded Reviewers (herein: Smellers):**

3 smellers will be chosen to participate in the study to perform a smell test using smell detector device; the "Nasal ranger" device, see the following link for technical information:

[http://www.nasalranger.com/Operations/Nasal%20Ranger%20Operations%20Manual%20v6.2.pdf](http://www.nasalranger.com/Operations/Nasal Ranger Operations Manual v6.2.pdf)

The Smellers will go through a short training in order to know how to operate the device and to assess their skills.

On day 0 pre procedure, the smellers will use the Nasal Ranger device to smell each of the 100 subject's genitals (baseline). Below are the room and procedure requirements. Only after the smelling procedure, will the subject undergo PrePex procedure.

The subjects will arrive to the clinic on days 3, 5 and 7 in order to document their odor levels.

On the Removal day (Day 7), the smellers will attend the Removal procedures and use the Nasal Ranger device. Below are the room and procedure requirements:

- 2 rooms will be used during the study which will have the same constant conditions (same room with no initial smell, same window, same distance from the penis: 30 cm)
- Usage of Nasal ranger device by trained personnel only
- The subjects will be randomized for their turn to enter the room
- When the smeller enters the room, the subject face will be covered and he will not wear his clothes but hospital garment
- After all 100 subjects were smelled by all 3 smellers, the subjects will be randomized again for their turn to enter the room (new randomization)
- When the smeller enters the room, the subject face will be covered and he will not wear his clothes but hospital garment
- The room should be aired between subjects
- The smelling will be always in the same distance from the genitals, by measuring with a ruler, 30 cm distance between the genitals and the end of the "Nasal Ranger"
- Each Smeller will be asked separately to grade the smell as he performs it as it is shown in the "Nasal ranger"
- Only at the end of the smelling procedure will the foreskin and device be removed.

**Questionnaire for Subject:**

The subject's own perception of the smell will be documented and graded in VAS of 1-10 and compared to his normal perception of his smell and of the environment perceptive as he sees it.

The subject will also be questioned about his compliance to the required hygiene procedure of his study arm.

**Questionnaire for partners:**

The perception of the subject's spouse/partner will be documented where possible, via a phone interview.

**PrePex Procedure:**

The subjects will go through a standard PrePex procedure which is described below.

The PrePex Operators and Assistants must be formally trained in the Rwanda PrePex Center of Excellence or by qualified PrePex trainers from Rwanda with the most up to date training program and certified as PrePex Operators and Assistants.

After assessment of eligibility and after the subject has signed the informed consent form, the subject will be enrolled in the study.

A **standard PrePex procedure** is described below:

The procedure will be carried out in a clean non-sterile environment. The subject will undergo sizing of the penis sulcus with the PrePex Sizing Plate. The penis will be cleaned by red Povidone-Iodine solution or other disinfection solution (Chlorahexidine, etc.) with the foreskin stretched backwards to expose the inner skin for the disinfection solution. Then the foreskin will be dried with a dry gauze. The Operator will then mark the circumcision line with a clean skin marker and then the Assistant will open the corresponding sized package of the PrePex device, and will load the Elastic Ring on to the Placement Ring. Then, Lidocaine 5% dermal cream will be applied on the inner foreskin. The Placement Ring with the Elastic Ring will be placed on the penis shaft and the foreskin tip will be stretched open to the sides by the operator hands, the operator may select to use a dry gauze to affirm the grip. Then, the Inner Ring will be applied through the stretched foreskin and over the glans penis (hereinafter, "glans") so that it will be placed fixed over the sulcus behind the glans (the base of the glans). At this point, the foreskin around the inner ring is held tight by the Assistant, and the Placement Ring and Elastic-Ring are approximated to the Inner Ring by the Operator. The foreskin is then adjusted to fit the circumcision marking line to the Elastic-Ring. Then, the Elastic-Ring is released from the Placement Ring on to the Inner Ring groove, initiating homeostasis. The procedure is then completed. Medication based on 400mg Ibuprofen is given to the subject to use at home if needed. The subject will then take part in a formal discharge session in a class room, where he will be instructed to return for a follow up visit and in any non sustained situation and to take painkillers in case of pain. The device will remain in situ for 7 days, at which the subject will return for a removal visit (i.e. if placed on Monday, removed on the following Monday). In case subjects arrive on day 5 or 6 and require removal of the device and foreskin, it is for the medical decision of the center managing physician to perform the removal if he/she thinks the removal is safe at this day. Those subjects will be followed for the procedure safety and efficacy. The foreskin is removed using sterile scissors, the Elastic Ring is removed using a #10 scalpel (surgical blade), and then the Inner Ring is extracted using a spatula or fingers, depending on the thickness of the foreskin. The area might be compressed with gauze for 30 seconds to avoid oozing and clear exudates. Following the removal process, the circumcision area will be dressed with a non-adherent sterile pad for 2 days, the subject will receive one dressing pad for home to change in case the dressing gets wet. The subject will then take part in a formal discharge session in a class room, where he will be instructed to turn to his local health center in any non sustained situation. Following the 2 days (day 9) the subject will remove the dressing on his own at home. The subject will be instructed to return in any non sustained situation, regardless of the follow-up visit.

**Follow-Up:**

The subjects will arrive for follow up visits on days 3, 5 & 7 in order to document the odor level.

There will be an interview phone call with the subject's partner (where possible) to assess hers perception of the smell. It will be conducted during days 5-6.

No follow up is required post Removal visit.

**Primary Objectives:**

- Safety - To determine the adverse events rate per foreskin cleaning technique per study arm
- Efficacy - To compare the different odor levels when examine 3 arms of patients undergoing PrePex circumcision with same personal hygiene and with different foreskin hygiene techniques

**Secondary Objectives**

- To determine an absolute odor level per each arm
- To determine a penis cleaning procedure guidelines safety and efficacy
- To determine the subject's perception of odor per study arm
- To compare the different perception of the subject’s partner relating to the odor when examine 3 arms of patients undergoing PrePex circumcision with same personal hygiene and with different foreskin hygiene techniques.

**Statistical Considerations:**

**Sample size:**

The intention is to obtain at least 100 evaluable subjects under Rwanda ethical responsibility (RNEC), determining to use the most deducted calculation setting and to maintain allocation ratio of 1:1:1 between the 3 study arms (33 per arm).

A sample size for the study was determined based on the two-sample t-test. In order to detect an effect size of 0.7 (Cohen's D) with 80% and at and a 5% level of significance between any two groups a sample size of 33 subjects is required. In the study each subject will have a smell test repeated by three different smellers this will be in order to examine the variability in odor sensation between different clinicians. Given this design and the three groups that will be compared, a statistically more powerful model (repeated measures ANOVA) is used instead of a t-test, and thus the study will be appropriately powered.

***Statistical Analysis:***

All data will be presented via descriptive statistics. Binary data such as adverse events success/fail criteria will be presented as a count and percentage together with an exact 95% confidence interval. Adverse event rates will be compared descriptively with the reported rates of similar treatments. Continuous data will be represented by a mean, standard deviation, median, minimum and maximum together with 95% confidence intervals for the means.

Study Flow

Screen 120 candidates

100 enrolled subjects

Randomization

Arm 1 -33 subjects

- Standard PrePex
- General hygiene instructions
- Foreskin hygiene general instructions – daily shower

Arm 2 -33 subjects

- Standard PrePex
- General hygiene instructions
- Foreskin specific hygiene instructions – daily soapy water with syringe
- **Follow up visits days 3, 5 & 7**
- Subject questionnaires
- Odor tests by 3 Blinded smellers
- Partner questionnaire – on days 3, 5 & 7
- Removal on day 7
- A random sample of subjects were evaluated a second time on day 7
- Safety - AEs

Arm 3 – 33 subjects

- Standard PrePex
- General hygiene instructions
- Foreskin specific hygiene instructions - daily chlorhexidine with syringe

|  | **Randomization & Screening Visit** | **Applying Visit** |  |  | **Removal Visit** |
| --- | --- | --- | --- | --- | --- |
| **Visit #** | **1** | **2** | **3** | **4** | **5** |
| **Day (D)/week (w)** | **–7 to D0** | **D0** | **D3** | **D5** | **D7** |
| Informed consent | X |  |  |  |  |
| Randomization | X |  |  |  |  |
| Inclusion/exclusion criteria | X |  |  |  |  |
| Medical history & Demography | X |  |  |  |  |
| Current medication including pain killers | X | X |  |  | X |
| Genital examination and potential pictures | X | X | X | X | X |
| Device apply |  | X |  |  |  |
| Foreskin Removal |  |  |  |  | X |
| Device Removal |  |  |  |  | X |
| Subject's subjective pain, tingling and discomfort evaluation |  | X | X | X | X |
| Expected Side effects and AE |  | X | X | X | X |
| Smellers test |  | X | X | X | X |
| Questionnaires for patients & for sub group female partners (between days 5-6) |  |  | X | X | X |
| Statistical analysis of data |  |  |  |  |  |

Table of Contents

[Study Synopsis 2](#__RefHeading___Toc344626056)

[Schedule of Events 8](#__RefHeading___Toc344626057)

[1. Clinical background 12](#__RefHeading___Toc344626058)

[2. STUDY OBJECTIVES AND PURPOSE 15](#__RefHeading___Toc344626059)

[2.1 Clinical Trial Design 15](#__RefHeading___Toc344626060)

[3. STUDY ENDPOINTS 15](#__RefHeading___Toc344626061)

[4. Selection and Withdrawal of Subjects 16](#__RefHeading___Toc344626062)

[4.1 Source of Subjects and Enrollment 16](#__RefHeading___Toc344626063)

[4.2 Eligibility Criteria 16](#__RefHeading___Toc344626064)

[*Subject Withdrawal Criteria* 17](#__RefHeading___Toc344626065)

[*Handling of Withdrawals* 17](#__RefHeading___Toc344626066)

[5. MATERIALS 18](#__RefHeading___Toc344626067)

[5.1 Product Name 18](#__RefHeading___Toc344626068)

[5.2 Intended Use 18](#__RefHeading___Toc344626069)

[5.3 Product Description 18](#__RefHeading___Toc344626070)

[5.4 Identification of the Investigational Medical Devices 18](#__RefHeading___Toc344626071)

[6. STUDY PROCEDURES – METHODS 18](#__RefHeading___Toc344626072)

[6.1 Study Location and Medical Team 18](#__RefHeading___Toc344626073)

[6.2 Informed Consent Procedure 19](#__RefHeading___Toc344626074)

[6.3 Study Schedule of Events 21](#__RefHeading___Toc344626075)

[6.4 Photography 22](#__RefHeading___Toc344626076)

[7. DeVICE MAINTENENCE AND DISPOSAL 23](#__RefHeading___Toc344626077)

[8. STUDY DESIGN 24](#__RefHeading___Toc344626078)

[8.1 Study Design 24](#__RefHeading___Toc344626079)

[8.2 Sample Size 24](#__RefHeading___Toc344626080)

[8.3 Statistical analysis 24](#__RefHeading___Toc344626081)

[8.3.1 General Considerations 25](#__RefHeading___Toc344626082)

[8.3.2 Study Populations 25](#__RefHeading___Toc344626083)

[8.3.3 Primary Endpoint Analysis 26](#__RefHeading___Toc344626084)

[9. ADVERSE EVENTS 26](#__RefHeading___Toc344626085)

[9.1 Definitions Used in This Study to Determine Severity of Adverse Event (AE) 26](#__RefHeading___Toc344626086)

[9.2 Definition of Adverse Event and Their Recording 27](#__RefHeading___Toc344626087)

[9.3 Definition of Serious Adverse Event (SAE) 27](#__RefHeading___Toc344626088)

[9.4 Guidelines to Determine the Relationship of an AE to the Study Device 28](#__RefHeading___Toc344626089)

[9.5 AE Regulatory Reporting Procedures 28](#__RefHeading___Toc344626090)

[9.6 Serious Unexpected Device Related Adverse Events Reported by the Sponsor (Expedited Safety Reports) 29](#__RefHeading___Toc344626091)

[9.7 Clinical Management of Study Related Adverse Events 29](#__RefHeading___Toc344626092)

[9.8 Criteria for Study Termination 30](#__RefHeading___Toc344626093)

[10. Study Monitoring 30](#__RefHeading___Toc344626094)

[10.1 Study Records/Source Document Inspection 30](#__RefHeading___Toc344626095)

[10.1 Study Records/Source Document Inspection 30](#__RefHeading___Toc344626096)

[10.2 Monitors 30](#__RefHeading___Toc344626097)

[10.3 Monitoring Plan 31](#__RefHeading___Toc344626098)

[11. Confidentiality/Publication of Study Results 32](#__RefHeading___Toc344626099)

[12. Records handling and Keeping 33](#__RefHeading___Toc344626100)

[12.1 Source Documents 33](#__RefHeading___Toc344626101)

[12.2 Data Collection Method 34](#__RefHeading___Toc344626102)

[13. REGULATORY OBLIGATIONS 34](#__RefHeading___Toc344626103)

[13.1 Sponsor's Obligations 34](#__RefHeading___Toc344626104)

[13.2 Investigator's Obligations 35](#__RefHeading___Toc344626105)

[14. Study Completion 36](#__RefHeading___Toc344626106)

[15. Protocol Deviations and Exceptions 36](#__RefHeading___Toc344626107)

[16. Removal of Subjects from the Study 36](#__RefHeading___Toc344626108)

[17. Ethical Conduct of the Study 37](#__RefHeading___Toc344626109)

[18. INVESTIGATOR AGREEMENT FOR PROTOCOL 38](#__RefHeading___Toc344626110)

[Reference 53](#__RefHeading___Toc344626111)

# Clinical background

In 2007 it was estimated that 33.2 million people were living with HIV and that there were 2.5 million new infections during that year. Discovering ways to prevent the transmission of HIV is of primary concern to health care authorities worldwide.

Numerous papers on the topic have been published over the past two decades to elevate HIV prevention awareness, especially in sub-Saharan countries.

In 2009, the US Government (USAID) reported that scaling up male circumcision (MC) to reach 80 percent of adult and newborn males in 14 African countries by 2015 could potentially avert more than 4 million adult HIV infections between 2009 and 2025 and yield annual cost savings of US$1.4 - 1.8 billion after 2015, with a total net savings of US$20.2 billion between 2009 and 2025.

To date, there are over 38 million adolescent and adult males in Africa that could benefit from MC for HIV prevention. The challenge Africa faces is how to safely scale up a surgical procedure in resource limited settings.

The PrePex device was accepted for the World Health Organization (WHO) list of prequalified MC devices and was listed on 31 May 2013.

PrePex became the first medical device for adult MC to receive WHO prequalification as an alternative to the conventional surgical circumcision methods already recognized by WHO.

Voluntary Medical Male Circumcision (VMMC) is a one-time, low cost intervention that shown to reduce men’s risk of HIV infection by approximately 70 percent. The WHO recommends VMMC as part of a comprehensive package of HIV prevention services. The WHO prequalification of PrePex represents an unprecedented opportunity to further the safe and rapid scale up of voluntary medical male circumcision programs [Official statement by the Office of the U.S. Global AIDS Coordinator].

Up to date more than 7,500 MCs were performed using the PrePex device. Rwanda, Zimbabwe and Uganda have completed the WHO clinical roadmap, scientifically validating the safety, efficacy, acceptability and superiority (over dorsal slit, on both measures of time and safety) of PrePex, other countries such as Kenya, Botswana and Mozambique are currently conducting PrePex pilot studies.

A concern that was raised by WHO TAG consulting members regarding the potential acceptability of PrePex by men involved the potential of strong odor extraction from the necrotizing foreskin. PrePex researchers from Rwanda have assumed that there is a possible relation of the level of odor to the personal hygiene and foreskin washing technique. It is speculated that when a patient follows an appropriate washing technique of the foreskin while wearing the device, the odor before device removal (day 7) will be redundant and significantly lower than the odor of the foreskin of a patient that does not follow such washing instructions and technique. Providing scientific evidence of the direct relation between odor and personal hygiene will allow VMMC implementing bodies to create a comprehensive and effective PrePex personal hygiene protocol and increase acceptability of the VMMC.

Rwanda has a national plan to offer a voluntary MC program to 2 million adult men in 2 years as part of a comprehensive HIV prevention strategy. To achieve this goal, the government launched a national study, based on the WHO Framework for Evaluation of Adult MC Devices, to assess the safety, efficacy and supremacy of the PrePex device when compared to surgical circumcision.

**Safety Study:**

The first safety study of PrePex was conducted in Rwanda between March 2010 to Dec 2010 in three steps: 5 subjects for pre-safety/feasibility, which went through self detachment of the foreskin and demonstrated the capability for foreskin self detachment; a pivotal phase of 50 subjects; a final extension phase of another 50 subjects.

A formal study report of the results of the first 40 subjects was accepted to CROI, a reputable infectious disease conference in Boston, USA. A report of the first pivotal study (full 50 subjects) was issued, the following represents a summary of the clinical experience.

**Study Summary:**

A study of 50 male subjects aged 18 to 35 years was conducted to assess the safety and efficacy of PrePex, a new medical device that has the potential to facilitate rapid scale-up of male circumcision (MC) in resource limited settings. The device is explored in the context of the Republic of Rwanda’s national scale-up plan, which calls for circumcision of 2 million adult men in 2 years as part of its national HIV prevention strategy.

The pivotal study was preempted by a feasibility stage that included 5 subjects. While this report focuses on the pivotal stage, it also offers insights from the feasibility study that lead to the methods and techniques in the pivotal stage.

The procedure was performed with no anesthesia. There were no cases of bleeding. There was no need for suturing. There were no clinical adverse events related to the procedure (from device Placement to device Removal) and there were no device-related incidents. Following device removal, there was 1 mild adverse event that was resolved with minimal intervention. Subjects returned to their daily routine shortly after Placement and immediately after removal of the device. The median time for complete healing[[1]](#footnote-2) was 21 days post device removal and the average was 25.3 days post device removal.

It was concluded that the tested device is safe. Furthermore, 100% of the subjects achieved the endpoint of complete circumcision, indicating device efficacy.

**Comparative Study:**

Background: This study was designed to compare a new non-surgical device with surgical MC, verifying the viability of the MC scale-up goal.

Methods: Prospective, randomized, controlled trial in Rwanda in which PrePex device was used for non-surgical MC and the dorsal-slit method (according to *WHO MC Manual v2.5C 08 MC under local anesthesia*) for surgical MC (ratio 2:1). Subjects were healthy adult male volunteers aged 21-54. The primary endpoint, set by WHO, was total MC procedure time.

Findings: Out of 217 eligible subjects, 144 randomized to PrePex/non-surgical arm and 73 to the surgical arm. All subjects were circumcised in 10 working days. Non-surgical MC was bloodless, without anesthesia, sutures or sterile setting, and with mean procedure time of 3.1 minutes skin to skin) - significantly shorter compared to mean surgical procedure time (15.4 minutes skin to skin) (p<0001). There were no device-related incidents.

Conclusion: PrePex MC is superior to surgical MC as measured by procedure time. Rwanda selected PrePex for national scale up.

**Cohort Nurses Study:**

Background: This study represents the third and final clinical evaluation of PrePex device, performed in mid 2011 in Rwanda, based on World Health Organization suggested framework for evaluating Male circumcision devices, following the validation of safety, efficacy and supremacy over surgical circumcision performed by surgeons. The national goal in Rwanda is to offer voluntary male circumcision to 2 million men within 2 years to decrease HIV incidence, and this can only be achieved if nurses can safely conduct the procedure, as there are not enough physicians. It was assumed that the Adverse Event rate will be below 2%.

Methods: Rwanda National Ethics Committee approved the study. The study was conducted in Rwanda Military Hospital, Kigali Rwanda, between July 2011 to September 2011. 10 nurses with no previous knowledge on PrePex device were trained for 3 days on PrePex circumcision method. 590 healthy adult male volunteers were enrolled in the study and distributed between 5 teams of 2 nurses each. The device works by stopping flow of blood to the distal foreskin, leading to necrosis of the tissue, which is removed, along with the device, after 7 days in situ. The Placement and Removal procedures were bloodless, required no injected anesthesia, no sutures and no sterile settings. AE data, severity and relation to device was gathered throughout weekly visit for 8 weeks follow up, post Removal.

Results: All 590 male subjects achieved the endpoint of complete circumcision with glans fully exposed. There were 2 device related moderate AE (rate of 0.34%) including 1 case of bleeding 6 hours post removal that required 1 suture and 1 case of erroneous placement that partially disturbed urination flow, and required replacement. There were 2 non-device related AE (rate of 0.34%), both moderate, including 1 subject self removal and 1 subject partial removal of the device, contrary to specific instructions. All AE were easily resolved with simple intervention.

Conclusions and Recommendations: The study statistically demonstrated that circumcision performed by nurses when using PrePex device is safe and effective. The procedure was bloodless and required no injected anesthesia, no sutures and no sterile settings (only standard consultation room). The device is officially approved for use in Rwanda. PrePex has the potential to facilitate rapid, safe, MC scale-up programs for HIV prevention by nurses, an imminent need in Sub Saharan Africa where physicians are a limited resource.

Overall, more than 5,900 PrePex procedures were conducted by June 2013 in Rwanda, where 1,900 procedures were performed in the frame work of clinical studies.

This clinical study will include the following objectives:

- To compare the different odor levels when examining 3 arms with identical personal hygiene but different foreskin hygiene techniques
- To determine a detailed and validated foreskin cleaning procedure protocol, that will ensure low odor without compromising safety

To compare the different perception of the subject’s partner relating to the odor when examining 3 arms with identical personal hygiene but different foreskin hygiene techniques.

This protocol describes the endpoints and methods of assessing those objectives.

# 2. STUDY OBJECTIVES AND PURPOSE

**Primary Objectives:**

- To compare the different odor levels when examine 3 arms of patients undergoing PrePex circumcision with same personal hygiene and with different foreskin hygiene techniques.

**Secondary Objectives**

- To determine an absolute odor level per each arm
- To determine a penis cleaning procedure guidelines safety and efficacy
- To compare the different perception of the subject’s partner relating to the odor when examine 3 arms of patients undergoing PrePex circumcision with same personal hygiene and with different foreskin hygiene techniques.

## 2.1 Clinical Trial Design

Assessing Odor Level when Using PrePex for HIV Prevention: A Prospective, Randomized, Open Label, Blinded Assessor Trial to Improve Uptake of Male Circumcision.

3. STUDY ENDPOINTS

**Endpoints:**

**Primary Endpoint:**

- The primary endpoint is to assess the different odor levels by evaluating 3 arms of patients with different foreskin hygiene techniques.

**Secondary Endpoints:**

- To determine the odor levels between 3 arms in order to assess patients' acceptability
- To determine a penis cleaning procedure guidelines safety and efficacy to be a part of the PrePex normal routine program
- To assess the patient's partner perspective of the smell to evaluate their acceptability.

4. Selection and Withdrawal of Subjects

## 4.1 Source of Subjects and Enrollment

Male subjects that are voluntary scheduled for circumcision. Only subjects who have signed the last version of an Independent Ethical Committee (IEC)/Institutional Review Board (IRB) approved Informed Consent Form (ICF) and meet all of the eligibility criteria listed below will be qualified for enrollment. The study will recruit at least 100 evaluable subjects who will complete the protocol procedure /treatment and the follow-up schedule.

## 4.2 Eligibility Criteria

**Eligibility Criteria:**

**Inclusion Criteria:**

- Ages – 21 to 49 years
- Subject wants to be circumcised
- Uncircumcised
- Able to understand the study procedures and requirements
- Agrees to participate in either arm and to follow hygiene instructions
- Agrees to have Independent blinded smell reviewers in the same room on Removal visit
- Agrees that his partner will be interviewed via telephone
- Agrees to abstain sexual intercourse for 6 weeks post device removal
- Agrees to abstain from masturbation for 2 weeks post device removal
- Agrees to return to the health care facility for follow-up visits (or as instructed) after his circumcision for a period of 1 week
- Subject able to comprehend and freely give informed consent for participation in this study and is considered by the investigator to have good compliance for the study
- Subject agrees to anonymous video and photographs of the procedure and follow up visits.

**Exclusion Criteria:**

- Active genital infection, anatomic abnormality or other condition, which in the opinion of the investigator prevents the subject from undergoing a circumcision
- Subject with the following diseases/conditions: phimosis, paraphimosis, warts under the prepuce, torn or tight frenulum, narrow prepuce, hypospadias, epispadias
- Known bleeding / coagulation abnormality, uncontrolled diabetes, by questionnaire
- Subject who have an abnormal penile anatomy or any penile diseases
- Subject that to the opinion of the investigator is not a good candidate
- Subject does not agree to anonymous video and photographs of the procedure and follow up visits.

## *Subject Withdrawal Criteria*

Subjects may be withdrawn from the study if any one or more of the following events occur:

- The subject wishes to withdraw from the study without providing any explanation
- Subject is lost to follow-up
- Refusal of the subject to continue treatment and/or follow-up observations
- Serious adverse event
- Significant protocol deviation/violation or noncompliance, either on the part of the subject or investigator
- Any decision made by the investigator or sponsor that termination is in the subject’s best medical interest
- Device failure or impossibility to continue surgical circumcision for any reason
- Other ethical or clinical considerations upon investigator discretion.

**NOTE: The subject must know in advance that if the subject wishes to withdraw from the study, he must undergo surgical circumcision immediately upon removal of the device.**

## *Handling of Withdrawals*

In accordance with the current revision of the Declaration of Helsinki and local Authority regulations at the corresponding countries, a subject has the right to withdraw from the study at any time, for any reason, without prejudice to his future medical care by the physician or the institution. The investigator and the sponsor also have the right to withdraw subjects from the study in the event of serious adverse events, protocol departure, or other reasons. Should a subject (or the subject’s legally authorized representative) decide to withdraw, all efforts will be made to collect and report the final visit observations, and the reasons for withdrawal, as thoroughly and timely as possible.

Withdrawals will be recorded, analyzed and reported to the local Ethical Committee.

5. MATERIALS

## 5.1 Product Name

PrePex

## 5.2 Intended Use

The PrePex device is intended to be used in a medical procedure to compress the foreskin of the penis to allow circumcision of an adult male.

## 5.3 Product Description

The PrePex device contains the following items:

1. A Placement Ring (O-Ring Introducer) made of medical grade biocompatible plastic polymer that is highly used in the medical device industry
2. An Inner Ring made of medical grade biocompatible plastic polymer
3. An O-Ring made of medical grade biocompatible elastic material
4. Verification thread

The use of the PrePex device should follow the Instruction for Use supplied by Circ MedTech and following an appropriate formal PrePex training course in a center of excellence located in Kigali, Rwanda, to the operator.

## 5.4 Identification of the Investigational Medical Devices

The label includes trade name and address of the manufacturer, batch code and all other essential information. Devices will be supplied clean and disinfected.

6. STUDY PROCEDURES – METHODS

## 6.1 Study Location and Medical Team

This protocol is submitted to Rwanda National Ethics Committee. The study center will be located in Rwanda Military Hospital.

## 6.2 Informed Consent Procedure

- - In obtaining and documenting informed consent, the investigator should comply with the applicable regulatory requirements, and should adhere to GCP, HIPAA and the ethical principles that have their origin in the Declaration of Helsinki
  - Prior to the beginning of the trial, the investigator should have the written informed consent form and any other written information approved by the National Ethical Committee before it is provided to subjects
  - The written informed consent form and any other written information to be provided to subjects should be revised whenever important new information becomes available that may be relevant to the subject’s consent. Any revised written informed consent form, and written information must be approved by the Ethic Committee before it is made available for subject signature. The subject should be informed in a timely manner if new information becomes available that may be relevant to the subject’s willingness to continue participation in the trial. The communication of this information should be documented
  - Neither the investigator, nor the trial staff, should coerce or unduly influence the subject to participate or to continue to participate in a trial. He will also testify of his agreement by a signature on the same ICF
  - The investigator, or a person designated by the investigator, should fully inform the subject of all pertinent aspects of the trial including the Ethical committee approved written informed consent
  - Before informed consent may be obtained, the investigator, or a person designated by the investigator, should provide the subject ample time and opportunity to inquire about details of the trial and to decide whether or not to participate in the trial. All questions about the trial should be answered to the satisfaction of the subject
  - Prior to a subject’s participation in the trial, the Ethical Committee approved written informed consent form should be signed and personally dated by the subject or by the subject's legally acceptable representative, and by the person who is authorized to conduct the informed consent discussion
  - Prior to participation in the trial, the subject should receive a copy of the signed and dated written informed consent form and any other written information provided to the subjects.
  - During a subject’s participation in the trial, the subject should receive a copy of the signed and dated consent form updates and a copy of any amendments to the written information provided to subjects.
  - Subject must provide informed consent and sign and date with his hand writing an Informed Consent Form (ICF) prior to any study related procedures being performed. The person reviewing the ICF with the subject must also sign and date the ICF. Each of the signers must sign and date the Form in the presence of the other signer.

**If subject is unable to comprehend and sign the Consent Form, subject must not be enrolled in the study.**

## 6.3 Study Schedule of Events

The following represents the study schedule of events

1. The procedure will be performed in Rwanda Military Hospital
2. **Inclusion Exclusion Criteria** – Subjects will be evaluated for inclusion in the study according to inclusion and exclusion criteria. Subject which do not meet the criteria will be offered circumcision out of the study procedures and records
3. **Informed Consent** – Potential candidate for enrollment to the study will be consented
4. **Randomization -** Subjects who meet all of the inclusion exclusion criteria will be randomly assigned to one of the study arms – Arm 1, Arm 2 or Arm 3

**Note -** The PrePex Operators and Assistants must be formally trained with the most up to date training program and certified as PrePex Operator and Assistants in a formal PrePex training program to take place in Kigali, Rwanda. In addition there may be training refreshment during the procedure of the first 20 procedures of the study.

1. **Circumcision** -

**Procedure:**

After assessment of eligibility and after the subject has signed the informed consent form, the subject will be enrolled in the study.

A **standard PrePex procedure** is described below:

The procedure will be carried out in a clean non-sterile environment. The subject will undergo sizing of the penis sulcus with the PrePex Sizing Plate. The penis will be cleaned by red Povidone-Iodine solution or other disinfection solution (Chlorahexidine, etc.) with the foreskin stretched backwards to expose the inner skin for the disinfection solution. Then the foreskin will be dried with a dry gauze. The Operator will then mark the circumcision line with a clean skin marker and then the Assistant will open the corresponding sized package of the PrePex device, and will load the Elastic Ring on to the Placement Ring. Then, Lidocaine 5% dermal cream will be applied on the inner foreskin. The Placement Ring with the Elastic Ring will be placed on the penis shaft and the foreskin tip will be stretched open to the sides by the operator hands, the operator may select to use a dry gauze to affirm the grip. Then, the Inner Ring will be applied through the stretched foreskin and over the glans penis (hereinafter, "glans") so that it will be placed fixed over the sulcus behind the glans (the base of the glans). At this point, the foreskin around the inner ring is held tight by the Assistant, and the Placement Ring and Elastic-Ring are approximated to the Inner Ring by the Operator. The foreskin is then adjusted to fit the circumcision marking line to the Elastic-Ring. Then, the Elastic-Ring is released from the Placement Ring on to the Inner Ring groove, initiating homeostasis. The procedure is then completed. Medication based on 400mg Ibuprofen is given to the subject to use at home if needed. The subject will then take part in a formal discharge session in a class room, where he will be instructed to return for a follow up visit and in any non sustained situation and to take painkillers in case of pain. The device will remain in situ for 7 days, at which the subject will return for a removal visit (i.e. if placed on Monday, removed on the following Monday). In case subjects arrive on day 5 or 6 and require removal of the device and foreskin, it is for the medical decision of the center managing physician to perform the removal if he/she thinks the removal is safe at this day. Those subjects will be followed for the procedure safety and efficacy. The foreskin is removed using sterile scissors, the Elastic Ring is removed using a #10 scalpel (surgical blade), and then the Inner Ring is extracted using a spatula or fingers, depending on the thickness of the foreskin. The area might be compressed with gauze for 30 seconds to avoid oozing and clear exudates. Following the removal process, the circumcision area will be dressed with a non-adherent sterile pad for 2 days, the subject will receive one dressing pad for home to change in case the dressing gets wet. The subject will then take part in a formal discharge session in a class room, where he will be instructed to turn to his local health center in any non sustained situation. Following the 2 days (day 9) the subject will remove the dressing on his own at home. The subject will be instructed to return in any non sustained situation, regardless of the follow-up visit.

**Follow-Up:**

The subjects will arrive for follow up visits on days 3, 5 & 7 in order to document the odor level.

There will be an interview phone call with the subject's partner (where possible) to assess hers perception of the smell. It will be conducted during days 5-6.

No follow up is required post Removal visit.

1. **Independent Blinded Reviewers (herein: Smellers):**

3 smellers will be chosen to participate in the study to perform a smell test using smell detector device; the "Nasal ranger" device, see the following link for technical information:

[http://www.nasalranger.com/Operations/Nasal%20Ranger%20Operations%20Manual%20v6.2.pdf](http://www.nasalranger.com/Operations/Nasal Ranger Operations Manual v6.2.pdf)

The Smellers will go through a short training in order to know how to operate the device and to assess their skills.

1. **Questionnaire for Subject:**

The subject's own perception of the smell will be documented and graded in VAS of 1-10 and compared to his normal perception of his smell and of the environment perceptive as he sees it.

The subject will also be questioned about his compliance to the required hygiene procedure of his study arm.

1. **Questionnaire for partners:**

The perception of the subject's spouse/partner will be documented where possible, via a phone interview.

## 6.4 Photography

**Photos:**

Photos of the Penis should be taken in each study visit according to the following guidelines:

- - The Camera must have a memory cards sufficient to contain the amount of expected photos of the largest file produced by the camera A spare set of new camera batteries must always be kept in the camera pack
  - A spare camera memory card must always be kept in the camera pack
  - Minimum of 2 photos, 1 from top and 1 from bottom of the glans must be taken presenting 360˚ around the glans and showing the frenulum. It is recommended to take 4 photos from 4 directions around the glans
  - Photos will be taken while a special photo frame is placed around the penis including the subject study number, date of photo, study day, and VAS pain value
  - Photos must be taken with a predefined Digital Camera
  - Camera settings must be on highest definition and resolution
  - Photos must be taken from a fixed distance with predefined lighting
  - Before releasing a subject photos must be verified for quality, specifically –
    - Focus of the photo on the skin surface
    - Lighting
    - Inclusion of subjects data in the photo

At the end of each photo day, all photos must be downloaded to a PC saved in a specific location and backed up on a removable Hard Drive. The Removable Hard Drive must be kept separate from the PC for ensuring no loss of data. Photos must never be erased from the camera digital memory card. In case the card is full a new card will be used and the full card will be saved in a secured location.

7. DeVICE MAINTENENCE AND DISPOSAL

The PrePex device is intended for single use only, device that was removed from the patient should be destroyed by a cutter before disposal to a bio-hazard box.

8. STUDY DESIGN

## 8.1 Study Design

Assessing Odor Level when Using PrePex for HIV Prevention: A Prospective, Randomized, Open Label, Blinded Assessor Trial to Improve Uptake of Male Circumcision.

## 8.2 Sample Size

One hundred (100) subjects scheduled for voluntary circumcision. The subjects will be randomly divided into three balanced study arms: Arm 1, Arm 2 and Arm 3 which will include thirty three (33) subjects respectively.

**Arm 1:** A control arm – subjects instructed to follow the current hygiene instructions of standard washing with soap and water during a daily shower. They will get a soap to take home and will be questioned daily to make sure they followed the cleaning instructions.

They might be requested to wash themselves at the hospital before the actual Removal visit starts.

**Arm 2:** subjects instructed to clean the foreskin with soapy water once a day using a syringe.

They will get a soap and required accessories to take back home and will be questioned daily to make sure they followed the instructions.

Subjects may be requested to wash themselves at the hospital before the actual Removal visit starts.

**Arm 3:** subjects instructed to clean the foreskin with diluted chlorhexidine (1%) once a day using a syringe. They will get chlorhexidine in tubes and required accessories to take back home and will be questioned daily to make sure they followed the instructions.

Subjects may be requested to wash themselves at the hospital before the actual Removal visit starts.

## 8.3 Statistical analysis

**Sample size:**

The intention is to obtain at least 100 evaluable subjects under Rwanda ethical responsibility (RNEC), determining to use the most deducted calculation setting and to maintain allocation ratio of 1:1:1 between the 3 study arms (33 per arm).

A sample size for the study was determined based on the two-sample t-test. In order to detect an effect size of 0.7 (Cohen's D) with 80% and at and a 5% level of significance between any two groups a sample size of 33 subjects is required. In the study each subject will have a smell test repeated by three different smellers this will be in order to examine the variability in odor sensation between different clinicians. Given this design and the three groups that will be compared, a statistically more powerful model (repeated measures ANOVA) is used instead of a t-test, and thus the study will be appropriately powered.

***Statistical Analysis:***

All data will be presented via descriptive statistics. Binary data such as adverse events success/fail criteria will be presented as a count and percentage together with an exact 95% confidence interval. Adverse event rates will be compared descriptively with the reported rates of similar treatments. Continuous data will be represented by a mean, standard deviation, median, minimum and maximum together with 95% confidence intervals for the means.

## 8.3.1 General Considerations

The primary analysis will be performed on an intent-to-treat basis. Per-protocol analysis also will be performed as a secondary analysis. The required significance level of findings will be equal to or lower than 5%. All statistical tests will be two-sided. Where confidence limits are appropriate, the confidence level will be 95%.

All data will be presented via descriptive statistics. Binary data such as adverse events success/fail criteria will be presented as a count and percentage together with an exact 95% confidence interval. Adverse event rates will be compared descriptively with the reported rates of similar treatments. Continuous data will be represented by a mean, standard deviation, median, minimum and maximum together with 95% confidence intervals for the means.

## 8.3.2 Study Populations

Male population between ages 21-49, resident in the center and outskirts of the Rwanda Military Hospital.

**Arm 1:** At least thirty three (33) adult men scheduled to undergo voluntary medical male circumcision (VMMC).

**Arm 2:** At least thirty three (33) adult men scheduled to undergo voluntary medical male circumcision (VMMC).

**Arm 3:** At least thirty three (33) adult men scheduled to undergo voluntary medical male circumcision (VMMC).

All scheduled for voluntary circumcision.

**8.3.2.1 Intent-to-Treat Population**

The intent-to-treat (ITT) population will include all patients enrolled in arms 1,2 or 3 of the study. This population will include all subjects from visit 1 that were placed with the device.

**8.3.2.2 Per-Protocol Population**

All subjects who complete the study without major protocol violations will be included in the per-protocol subset.

## 8.3.3 Primary Endpoint Analysis

Baseline demographics and patient characteristics will be presented in tabular format. The statistical evaluation of baseline characteristics will include all available data from the ITT population.

The analysis of odor levels will include database which will analyze the correlation between odor levels, subjects and subjects' partners questionnaire results.

The primary end-point evaluations will be performed based on both the ITT and the per-protocol populations.

# ADVERSE EVENTS

## 9.1 Definitions Used in This Study to Determine Severity of Adverse Event (AE)

- Mild – Event that is noticeable but transient, requires no treatment, and does not interfere with the subject’s daily activities.
- Moderate – Sign or symptom which may be ameliorated by simple therapeutic measures: may interfere with usual activity.
- Severe - Sign or symptom that is intense or debilitating and that interferes with usual activities. Recovery is usually aided by therapeutic measures and the discontinuation of the study device may be required.

## 9.2 Definition of Adverse Event and Their Recording

AE definition: Any unfavorable and unintended sign, symptom or disease temporally associated with the use of a medical treatment and/or procedure regardless of whether it is considered related to the medical treatment/procedure (attribution of unrelated, unlikely, possible, probable, or definite).

**In this study, all expected side effects and device related or unrelated AE will be recorded in the CRF.**

The nomenclature for AE should be recorded as in the CTCAEv3 and according to the PrePex MC Classification of Adverse Events and Device Hazards.

NOTE – There may be expected procedure side effects which are not considered as Adverse

Events and will not be included in the primary safety analysis, those include:

- - Localized Edema
  - Oozing
  - Clear exudates
  - Slough (indicators: white caramel tissue over the exposed granulating tissue, may appear on day 9)

## 9.3 Definition of Serious Adverse Event (SAE)

Any adverse event experience that results in any of the following outcomes

- Death
- A life threatening event
- Requires or prolongs inpatient hospitalization or re hospitalization
- Persistent or significant disability/incapacity
- A congenital anomaly or birth defect
- Medical or surgical intervention to avoid impairment of body function

## 9.4 Guidelines to Determine the Relationship of an AE to the Study Device

- Definite: The adverse event is clearly related to the investigational treatment.
- Probable: The adverse event is likely related to the investigational treatment.
- Possible: The adverse event may be related to the investigational treatment.
- Unlikely: The adverse event is doubtfully related to the investigation treatment.
- Unrelated: The adverse event is clearly NOT related to the investigational agent(s).

## 9.5 AE Regulatory Reporting Procedures

- Any clinical study event that is judged to be a device related AE should be recorded on the Case Report Forms (CRFs) AE pages during the course of the study, as detailed in section 9.2 above. The Principal Investigator (PI) (or designee) and/or study coordinator should ensure that this information will be captured during every study subject visit. Any information that is recorded in the CRF must rely on records in the source document. In this study working sheets are allowed as source documents.
- Whenever a study subject has reported **any** device related AE, the study coordinator or PI designee will discuss the event as soon as possible with the PI (if possible while the study subject is there). The PI must evaluate the event.
- If the AE is not serious, the information will be recorded on the appropriate CRF AE Form as instructed above. The AE will be managed medically as appropriate, and will be followed until resolution.
- Any SAE (according to the definitions above), whether or not related to the investigational product, must be reported by the PI or designee simultaneously to the sponsor’s representative, to the Ethical Committee and to the board immediately (within **24 hours** after learning of the event) for accurate and timely recording and reporting by the CRF's SAE Form and managed medically as appropriate, and will be followed until resolution. At intervals per PI discretion, a follow-up CRF SAE Form will be filled and sent to the sponsor's representative. Any relevant information available on the event (hospital records, lab tests, discharge summaries, etc.) will be applied to determine whether the SAE is Unexpected AND related to the investigational product and requires reporting the event to the Ethical Committee and to the MOH ("Expedited Safety Report", see section 9.6 ahead). Any additional information on the SAE should be also forwarded to the sponsor’s representative for further follow-up on special SAE follow-up forms.

Each SAE will be summarized in a form of a "SAE Report".

- **All SAE Reports shall be sent to the Ethics Committee periodically, unless in the following incidences:**

**- Within 48 hours of learning of event occurrence in case of any unexpected** **and device related** **SAE**.

- For reasons of confidentiality, subject identifying data (e.g., name, social security numbers) should not be disclosed outside the hospital whatsoever. Instead, the study-specific code number should be used.

Sponsor representatives will provide direction for consistent and systematic handling of such reports and determine their responsibility to the Health Authority as stipulated by applicable MDR regulations. Only **Investigational Product Related and Unexpected SAE’s** will be reported to the Health Authorities. Therefore, not all SAEs/Product Complaints reported to Sponsor will require subsequent notification to the Health Authority. However study investigators will be informed by clinical study personnel of any serious adverse event and product complaint reported to the Health Authority associated/related to the medical device used in this study.

Sponsor clinical and medical teams will review all **unexpected and related SAE’s** to evaluate them for further recommendation of study status, as well as other device related AE incidence evaluation.

## 9.6 Serious Unexpected Device Related Adverse Events Reported by the Sponsor (Expedited Safety Reports)

All Serious Unexpected Device Related Adverse Events reports that will be issued by Sponsor will be forwarded as "Expedited Safety Reports" to the PIs. Each PI will send these expedited safety reports to the ETHICS COMMITTEE (the PI in each site should have notify the ETHICS COMMITTEE of such event 48 hours after learning of the event as mentioned above, not in a form of "Expedited Safety Report"). Sponsor will send such reports to the MOH as required the international and national guidelines. The PI and/or study coordinator will file copies of these safety reports in the regulatory binder of the site file.

## 9.7 Clinical Management of Study Related Adverse Events

AEs will be clinically managed according to medical indications, and the standard of care in the specific Medical Center.

Subjects will be entitled for continuation of clinical follow-up and treatment until of their study related clinical problem has been resolved.

## 9.8 Criteria for Study Termination

Termination of the study may be due to any of the following reasons:

- Device related SAEs which are life threatening in at least two cases.
- Device failures in more than 5% of the cases.
- Other ethical or clinical considerations
- Decision by the investigator or sponsor that termination is in the subjects’ best medical interest

# Study Monitoring

## 10.1 Study Records/Source Document Inspection

The investigator will allow the research team (nurses, data managers etc.), representatives of Sponsor, its monitoring team, the MOH of Rwanda, and other governmental regulatory agencies to monitor/audit/inspect all study records, CRFs, IRB/IEC records, and corresponding portions of the subject’s office and/or hospital medical records at regular intervals throughout the study. These monitoring/audits/inspections are conducted to verify adherence to the protocol, integrity of the data being captured on the CRFs and compliance with applicable regulations. Sponsor obliged that subjects’ medical records will be maintained in a confidential manner. Study reports will not identify subjects by name or other identifiers.

## 10.2 Monitors

Each study's site will be monitored by a qualified representative of the Sponsor or any qualified monitor delegated to do so, on behalf of the Sponsor to monitor each study subject’s data and study conduct at regular intervals throughout the course of the study according to a pre-defined monitoring plan. On site monitoring of the investigator’s facilities aids in ensuring compliance with the protocol.

Any deficiency noted during the monitoring visits will be discussed with the investigator and the corrective actions to be taken agreed upon. Should the sponsor determine, at any time during the study that the investigator is not in regulatory and/or protocol compliance, measures necessary to establish compliance will be implemented. If compliance cannot be maintained, the sponsor will suspend or terminate the study at this site.

## 10.3 Monitoring Plan

***I. Pre-Study Visit***

1. Assess the site’s infrastructure (staff and facility) for the capability to conduct the study.
2. Evaluate Investigator and staff’s Experience, Qualifications and Capabilities- signed and dated CVs.
3. Financial Disclosure information.

The sponsor may waive the pre-study visit in certain sites based on previous knowledge or experience with the site or personnel, or other reasons as deemed acceptable.

***II. Study Initiation Visit***

Orients the investigator’s staff involved in the study on

1. Protocol content and procedures
2. CRF and fill-in process including queries resolution process
3. GCP and other regulatory requirements
4. Informed Consent form and process
5. AE, SAE, Safety reporting
6. Ethical Committee
7. Investigational product accountability
8. Subject information- subject’s identification log, subject pre-screening and screening logs, subject enrollment log, subject study visit log
9. Study monitoring
10. Investigator’s site file (ISF)
11. Report of AE back from the Sponsor to the sites (Expedited Safety reports)
12. Expectations from the site regarding data collection and timelines
13. Ministry of health

The sponsor may choose to perform a regional Investigator Meeting instead of the initiation

visits at each site.

***III. Regular Monitoring Visits***

1. Check on the progress of the study
2. Protocol and GCP compliance
3. Informed Consent form and process
4. CRF completion, correction, source data verification
5. AE, SAE, Safety reporting
6. Investigational product
7. Investigator’s study file
8. Pictures and videos

Detailed monitoring schedule to be provided at study initiation

***IV. Final Study Visit - Close Out Monitoring Visit***

Ensure Investigator understands the on-going responsibilities

1. Record archival practices for source documents and CRFs after completion of the study - up to 15 years.
2. Follow-up of on-going device related adverse events - up to 30 days after completion of the study and of SAE – until resolution.
3. Notify the sponsor in the event of Health Authority inspection.
4. Finalizing all "open" issues and complete Source Data Verification (SDV).

# Confidentiality/Publication of Study Results

This clinical study is confidential and should not be discussed with individuals outside the study. Additionally, the information in this document and in the study may contain secrets and commercially sensitive information that is confidential and may not be disclosed unless such disclosure is required by federal or state law or regulations. Subject to the foregoing, this information may be disclosed only to those persons involved in the study that have a need to know, but all such persons must be instructed not to further disseminate this information to others.

The data may be used now and in the future for presentation or publication at the investigator and/or sponsor’s discretion or for submission to governmental regulatory agencies.

All reports and communications relating to subjects in the study will identify each subject only by the subject’s initials and by the subject’s study number.

# Records handling and Keeping

## 12.1 Source Documents

Source documents are the initial documents whereon subject data are recorded. This includes, but is not limited to, original subject files, hospital records, and original recordings/tracings from automated instruments, etc.

The sponsor allows securing dedicated work sheets to serve as source documents to collect the available source data, on top of all other applicable source documents in the site.

All information captured on the CRF should be accurately supported by the source documents unless specifically approved and documented by Sponsor.

For example, each subject’s source documents should include (but not be limited to):

- Documenting the Informed consent process
- Subject full name and identification
- Date of each study required visit with a description of the visit and the results of each procedure that was performed
- A full and comprehensive anamnesis that will cover subject's medical history, current disease etc.
- All concomitant procedures and medications for the screening eligibility purposes and regular visits

Any additional information relevant to the study should be included in the subject’s source documents. In particular, any deviations from the study protocol or procedures should be recorded in the source documents, if noted. For example, if study required procedures or visits are not completed or are completed outside the time frame specified in the protocol, the reasons for the departure should be explained in the source documents, or mentioned whether it was waived by the Sponsor in advance. The investigator must maintain all study documentation at least 2 years after the last approval of marketing application and until there are no pending or contemplated marketing application or at least 2 years have elapsed since the formal discontinuation of clinical development of the investigational product. These documents should be retained for a longer period, however, if required by the applicable regulatory requirements or by an agreement with the sponsor. The sponsor should inform the investigator in writing when the trial-related records are no longer needed.

## 12.2 Data Collection Method

Data from the subject’s permanent medical records (see source documentation section) will be recorded on CRFs supplied by the sponsor. These CRFs will be used to transmit the information collected in the performance of this study to the clinical database either manually or by site data entry (a third party vendor may be used for this). The CRF for this study will be of standard type with 1 original and a copy will be created, in which the original copy will be kept on site.

Corresponding CRFs should be completed immediately after each subject's visit.

All source data must be typewritten or filled out in ball pens, accurately and promptly following each examination or surgery. The corresponding CRF should be completed that no fields will be left blank. CRFs entries corrections will be made only by crossing out (single line) incorrect data and writing in the revisions. All corrections must be initialed and dated by the individual performing/recording them. If the reason for the change is not obvious, an explanation will be recorded. Blacking out or using correction fluid or an eraser is not allowed to eliminate data. The investigator must review the CRFs for completeness and accuracy and must sign/date the forms where indicated. Signature stamps or substitutes are not acceptable. The investigator will retain originals of all source documents, subject consent forms, and study data as a permanent record.

Each set of CRFs copy should be reviewed for accuracy and completion (signatures, dates, adverse events, serious adverse events, protocol departures) and maintained in the investigator’s study site.

# 13. REGULATORY OBLIGATIONS

## 13.1 Sponsor's Obligations

Clinical research studies are subject to the Rwanda National Ethics Committee approval. A Sponsor must assume the following responsibilities and must keep the required records. He/she must:

1. Provide the Investigator with the necessary information- Protocol and Device User's Manual or Instructions for Use (maybe incorporated to the Investigator Brochure)
2. Inform the Investigator of all new information that may affect his/her decision of whether to continue their participation in the study
3. Provide the supplies (PrePex device and additional materials) for the investigation
4. Provide source to purchase appropriate non-adherent pads for dressing

Maintain the following records:

1. A signed protocol and relevant documents
2. All correspondence that relate to the clinical trial
3. Signed Investigator Agreement
4. Records of device shipment and disposal (shipping receipts, material destruction records, etc.)
5. Other records as required by the Health Authority.

## Investigator's Obligations

Clinical research studies are subject to the regulations of the Regulatory Authority of the country. Upon signing the protocol, the Investigator agrees to assume the following responsibilities and to keep the required records for a period of three years following completion of the study and to file the required reports in a timely manner:

1. Conduct the Investigation in compliance with the protocol. Changes to the protocol may only be made after approval by the Sponsor and the Ethical Committee, or when necessary to protect the safety, rights or welfare of a subject
2. Personally conduct or supervise the investigation
3. Read and understand the information in the Protocol and Investigator Brochure
4. Be aware of the potential risks and side effects of the PrePex device
5. Ensure that all associates, colleagues and employees assisting in the conduct of the study are informed about their obligations
6. Inform all subjects that the device is being used for investigational purposes and ensure that the requirements related to obtaining the informed consent are met
7. Dispose or return of remaining supplies as directed by the Sponsor.

Maintain the following records (for a period of three years following completion of the study):

1. Signed copy of the protocol
2. Signed consent forms
3. All correspondence as relates to the clinical trial
4. Case Report Forms.

File the following reports:

1. Serious unexpected adverse device related effects reports received by the sponsor
2. Regular Serious Adverse Event reports produced by the PI
3. Deviations, which were made from the protocol for emergency use, must be reported to the sponsor as soon as possible, but no later than five working days after its occurrence.

# 14. Study Completion

The PI will complete and report the study in satisfactory compliance with the protocol.

It is agreed that, for any reasonable cause, either the PI or the Sponsor, may terminate this study, provided a written notice is submitted at a reasonable time in advance of intended termination. If the study is terminated for safety reasons, the investigator will be notified immediately by telephone, followed by written instructions for study termination notification of the Ethical Committee.

# 15. Protocol Deviations and Exceptions

The investigator should not implement any deviation from, or changes of, the protocol without agreement with the sponsor and prior review and documented approval from the Ethical Committee of an amendment, except where necessary to eliminate an immediate hazard(s) to trial subjects.

The investigator should document and explain any deviation from the approved protocol and to file waivers received from the sponsor, if applicable. The reasons for it, and, if appropriate, the proposed protocol amendments should be submitted to:

1. The Sponsor for agreement
2. Ethical Committee
3. The Board
4. The regulatory authority

# 16. Removal of Subjects from the Study

A subject has the right to withdraw from the study at any time, for any reason, without prejudice his future medical care by the physician or the institution. Should a subject decide to withdraw, all efforts will be made to collect and report the final visit observations as thoroughly and timely as possible. **However,** **subject should know that if the subject wishes to withdraw, he will need to undergo surgical circumcision.**

Subjects may be removed from the study if any one or more of the following events occur:

- Refusal of the subject to continue treatment and/or observations;
- Decision by the Investigator that termination is in the subject’s best medical interest;
- Subject is lost to follow-up;
- Other ethical or clinical considerations.

# 17. Ethical Conduct of the Study

This study will be conducted in compliance with the protocol after approval of the local Ethical committee, and according to Good Clinical Practice (GCP) and international standards such as ISO 14155.

No deviation from the protocol, after sponsor's approval will be implemented without the prior review and approval of the Ethical Committee except where it may be necessary to eliminate an immediate hazard to a research subject. In such case, the deviation will be reported to the Ethical Committee and to the Board as soon as possible.

A copy of the protocol, Informed Consent Form (ICF), advertising material and all documents handed out to the subjects, must be submitted to the Ethical Committee. Written approval of the protocol Informed Consent Form, advertising material and documents handed out to the subjects, must be obtained prior to subject enrollment by the Ethical Committee.

# 18. INVESTIGATOR AGREEMENT FOR PROTOCOL

I have read the foregoing protocol

"Assessing Odor Level when Using PrePex for HIV Prevention: A Prospective, Randomized, Open Label, Blinded Assessor Trial to Improve Uptake of Male Circumcision" and agree to:

- Conduct the study as outlined herein;
- Maintain the confidentiality of all information received or developed in connection with this protocol and
- Conduct this study in accordance with GCP Standards and any other applicable local/state laws and regulations;
- Comply with the signed investigators agreement.

__________________________________ __________________________

**Investigator Signature Date**

**______________________________________________________________________**

**Investigator name in capital letters**

**______________________________________________________________________**

**Telephone number:**

**______________________________________________________________________**

**Fax number:**

**______________________________________________________________________**

**Email address:**

APPENDIX 1. PrePex MC Classification of Adverse Events and Device Hazards

Rev - 19^th^ June 2012

**PrePex MC**

**Classification of Adverse Events and Device Hazards**

| **Adverse**  **Event** | **Description** | **Severity** | **Code** |
| --- | --- | --- | --- |
| **A. During Placement** | | | |
| Pain | Pain score of 8 or more not requiring anesthesia  Requires anesthesia  Not controlled by additional anesthesia | Mild  Moderate  Severe | APP1  APP2  APP3 |
| Difficulty in applying the device | Could not apply device; determined as contra indicated; no harm to tissue or subject  Had to push unusually hard, but no harm to tissue or subject and no change to procedure  The device cut through the foreskin, with or without minor bleeding, No change in procedure  The device cut through the foreskin, with or without minor bleeding, and requiring a change to surgical method  The device cut through the foreskin, causing significant bleeding and requiring a change to surgical method | No AE  No AE  Mild  Moderate  Severe | --  --  ADD1  ADD2  ADD3 |

| **Adverse**  **Event** | **Description** | **Severity** | **Code** |
| --- | --- | --- | --- |
| **B. While Wearing the Device** | | | |
| Pain / Discomfort | Not requiring intervention beyond painkiller or anesthetic cream  Requiring early device removal by PrePex Operator or by client  Requiring early device removal and anesthesia  Not controlled by device removal or additional anesthesia | Not AE  Mild  Moderate  Severe | --  BWP1  BWP2  BWP3 |
| Device displacement / spontaneous detachment | Device displacement with no clinical consequences  Complete spontaneous detachment, or patient removed device himself with no adverse clinical consequences  Device displacement requiring re-placement  Device displacement requiring surgical intervention  Displacement or detachment and penile damage present | Not AE  Mild  Moderate  Severe | --  BDD1  BDD2  BDD3 |
| Early device removal (i.e. 4 days or less with the device) | Device removed due to pain, swelling or bleeding  Device removed due to pain, swelling or bleeding requiring surgical intervention  Device removed and penile damage present | Mild  Moderate  Severe | BER1  BER2  BER3 |
| Edema | More edema than usual but not causing any discomfort to the patient  Moderate edema causing the patient discomfort, though managed with conservative measures  Severe edema, causing the patient discomfort, uncontrolled with conservative measures | Mild  Moderate  Severe | BED1  BED2  BED3 |
| Hematoma | Mild contained hematoma, not requiring any treatment  Hematoma requiring surgical drainage/exploration but no evidence of active bleeding  Rapidly expanding hematoma suggesting active bleeding requiring surgical exploration or referral | Mild  Moderate  Severe | BHM1  BHM2  BHM3 |

| **Adverse**  **Event** | **Description** | **Severity** | **Code** |
| --- | --- | --- | --- |
| **C. During Device Removal** | | | |
| Pain | Pain score of 6 or less lasting for less than 2 minutes  Pain score of 8 or more lasting for over 2 minutes  Requires anesthesia  Not controlled by additional anesthesia | Not AE  Mild  Moderate  Severe | --  CPR1  CPR2  CPR3 |
| Excessive bleeding | More bleeding than usual, but easily controlled  Bleeding that requires suture to control  Blood transfusion or transfer to another facility for management required | Mild  Moderate  Severe | CBL1  CBL2  CBL3 |
| Edema | More edema than usual but not causing any discomfort to the patient  Moderate edema causing the patient discomfort, though managed with conservative measures  Severe edema, causing the patient discomfort, uncontrolled with conservative measures | Mild  Moderate  Severe | CED1  CED2  CED3 |
| Hematoma | Mild contained hematoma, not requiring any treatment  Hematoma requiring surgical drainage/exploration but no evidence of active bleeding  Rapidly expanding hematoma suggesting active bleeding requiring surgical exploration or referral | Mild  Moderate  Severe | CHM1  CHM2  CHM3 |
| Infection | Pain and erythema with no obvious swelling  Painful swelling with erythema or elevated temperature or purulent wound discharge  Cellulitis or wound necrosis | Mild  Moderate  Severe | CIN1  CIN2  CIN3 |
| Device removal difficulties | Difficult removal, with pain score of 8 or more lasting for over 2 minutes  Difficult removal, with abrasion of shaft or glans  Difficult removal, requiring injection of local anesthetic or requiring up to three sutures post-removal  Difficult removal, requiring more than three sutures  Difficult removal, with penile damage | Mild  Mild  Mild  Moderate  Severe | CDR1.1  CDR1.2  CDR1.3  CDR2  CDR3 |
| Damage to the penis | Mild bruising or abrasion, not requiring treatment  Bruise or abrasion to the glans or shaft of the penis requiring pressure dressing or surgery to control  Portion or all of the glans or shaft of the penis severed | Mild  Moderate  Severe | CDP1  CDP2  CDP3 |

| **Adverse**  **Event** | **Description** | **Severity** | **Code** |
| --- | --- | --- | --- |
| **D. Within 6 Weeks Post Removal** | | | |
| Pain | Symptoms of pain requiring bed rest for less than half the day  Pain requiring bed rest for more than half day  Excruciating pain requiring total bed rest | Mild  Moderate  Severe | DPA1  DPA2  DPA3 |
| Excessive bleeding | More bleeding than usual, but easily controlled  Bleeding that requires suture to control  Blood transfusion or transfer to another facility for management required | Mild  Moderate  Severe | DBL1  DBL2  DBL3 |
| Edema | More edema than usual but not causing any discomfort to the patient  Moderate edema causing the patient discomfort, though managed with conservative measures  Severe edema, causing the patient discomfort, uncontrolled with conservative measures | Mild  Moderate  Severe | DED1  DED2  DED3 |
| Hematoma | Mild contained hematoma, not requiring any treatment  Hematoma requiring surgical drainage/exploration but no evidence of active bleeding  Rapidly expanding hematoma suggesting active bleeding requiring surgical exploration or referral | Mild  Moderate  Severe | DHM1  DHM2  DHM3 |
| Infection | Pain and erythema with no obvious swelling  Painful swelling with erythema or elevated temperature or purulent wound discharge  Cellulitis or wound necrosis | Mild  Moderate  Severe | DIN1  DIN2  DIN3 |
| Damage to the penis | Mild bruising or abrasion, not requiring treatment  Bruise or abrasion to the glans or shaft of the penis requiring suture or surgery to control  Portion or all of the glans or shaft of the penis severed | Mild  Moderate  Severe | DDP1  DDP2  DDP3 |
| Delayed wound healing | Healing takes longer than usual, but no extra treatment necessary  Additional non-operative treatment required  Requires re-operation to correct | Mild  Moderate  Severe | DDW1  DDW2  DDW3 |
| Appearance | When healing is complete, subject concerned, but no discernable deformity  When healing is complete, minimal deformity does not require re-operation  Significant deformity requires re-operation to correct | Mild  Moderate  Severe | DAP1  DAP2  DAP3 |
| Problems with voiding | Transient complaint by subject that resolves without treatment  Requires a special return to the clinic, but no additional treatment  Requires referral to another facility for management | Mild  Moderate  Severe | DVO1  DVO2  DVO3 |

| **Adverse**  **Event** | **Description** | **Severity** | **Code** |
| --- | --- | --- | --- |
| **E. Six Weeks or More Post Removal** | | | |
| Infection | Pain and erythema with no obvious swelling  Painful swelling with erythema or elevated temperature or purulent wound discharge  Cellulitis or wound necrosis | Mild  Moderate  Severe | EIN1  EIN2  EIN3 |
| Delayed wound healing | Healing takes longer than usual, but no extra treatment necessary  Additional non-operative treatment required  Requires re-operation to correct | Mild  Moderate  Severe | EDW1  EDW2  EDW3 |
| Appearance | Subject concerned, but no discernable deformity  Minimal deformity does not require re-operation  Significant deformity requires re-operation to correct | Mild  Moderate  Severe | EAP1  EAP2  EAP3 |
| Excessive skin removed | Client concerned, but there is no deformity on erection  Causes slight discomfort on erection but surgical correction not necessary  Interferes with sexual life and surgical correction is necessary | Mild  Moderate  Severe | EES1  EES2  EES3 |
| Insufficient skin removed | Prepuce partially covers the glans only when extended  Prepuce still partially covers the glans and re-operation is required to correct  Not applicable | Mild  Moderate  Severe | EIS1  EIS2  EIS3 |
| Torsion of penis | Torsion is observable, but does not cause pain or discomfort  Causes mild pain or discomfort on erection, but additional operative work not necessary  Requires re-operation or transfer to another facility to correct the problem | Mild  Moderate  Severe | ETP1  ETP2  ETP3 |
| Erectile dysfunction | Client reports occasional inability to have an erection  Client reports frequent inability to have an erection  Client reports complete or near complete inability to have erections | Mild  Moderate  Severe | EED1  EED2  EED3 |
| Psycho-behavioural problems | Client reports mild sexual dissatisfaction attributed to male circumcision, but no significant psycho-behavioral consequences  Client reports significant sexual dissatisfaction attributed to male circumcision, but no significant psycho-behavioral consequences  Significant depression or other psychological problems attributed by the participant to the male circumcision | Mild  Moderate  Severe | EPB1  EPB2  EPB3 |
| Other AEs | Other AEs are described below* | -- |  |

**Other AEs**

- Liver or pancreatic abnormalities;
- Neurologic conditions of the central nervous system (e.g., meningitis, encephalitis, convulsions and headaches, visual and auditory disturbances, strokes), and peripheral neurologic conditions (e.g., peripheral neuropathies, motor weakness etc.);
- Myocarditis and pericarditis;
- Dermatologic condition affecting the genital, pubic or perianal areas or groin; and,
- Injuries and accidents.

Common illnesses unrelated to male circumcision will not be included in the “other AE” category to avoid unnecessary reporting of irrelevant events. Such AEs include the following:

- Malaria and other common parasitic infections;
- Gastrointestinal (GI) tract diseases, including diarrhea, gastroenteritis (bacterial, viral or parasitic), and other GI conditions, and oropharangeal infections;
- Respiratory illnesses (Upper Respiratory Tract Infections, acute lower respiratory infection, pleurisy), including tuberculosis, influenza or other respiratory infections;
- Angina, myocardial infarction, congestive cardiac failure;
- Dermatologic conditions (e.g., scabies, infected and uninfected rashes, pruritis, fungal infection), except those affecting the genital, pubic or perianal areas or groin
- Neoplasms not affecting the genitourinary tract; and,
- Surgical conditions not related to the intervention (e.g., hernias) and which do not require hospitalization.

2. APPENDIX A. REFERENCES

**Articles from journals and medical publications including World Health Organziation**

Anonymous. Newer approaches to HIV prevention*. Lancet* 2007;369(9562):615 (Editorial).

Auvert B, Taljaard D, Lagarde E, Sobngwi-Tambekou J, Sitta R, Puren A. Randomized, controlled intervention trial of male circumcision for reduction of HIV infection risk: the ANRS 1265 Trial. *Public Library of Science Medicine* 2005;2(11):e298. Erratum in: *PLoS Med*. 2006;3(5):e298.

Bailey R, Moses S, Parker CB, et al. Male circumcision for HIV prevention in Kisumu, Kenya: A randomized controlled trial. *Lance*t 2007;369:643-56.

Bailey RC. *Evaluation of a trial intervention integrating male hygiene and circumcision with reproductive health in Nyanza Province.* Unpublished report to AIDSMARK; 2002.

Bailey RC. *Needs assessment: Introduction of male circumcision services for HIV prevention in Kisumu and Suba districts. Kenya, funded by US CDC (Kenya), PEPFAR and CHF International*. 2006.

Bailey RC, Egesah O, Rosenberg S. Male circumcision for HIV prevention: a prospective study of complications in clinical and traditional settings in Bungoma, Kenya. *Bull WHO* 2008;9:669-77.

Bailey RC, Muga R, Poulussen R, Abicht H. The acceptability of male circumcision to reduce

HIV infections in Nyanza Province, Kenya. *AIDS Care* 2002;14(1):27-40.

Bailey RC, Neema S, Othieno R. Sexual behaviors and other HIV risk factors in circumcised and uncircumcised men in Uganda. *Journal of Acquired Immune Deficiency Syndromes* 1999;22(3):294-301.

Gray RH, Kigozi G, Serwadda D, et al. Male circumcision for HIV prevention in men in Rakai,

Uganda: a randomised trial. *Lancet* 2007;369(9562):657-66.

Halperin DT, Fritz K, McFarland W, Woelk G. Acceptability of adult male circumcision for sexually transmitted disease and HIV prevention in Zimbabwe. *Sexually Transmitted Diseases* 2005;32(4):238-9.

Kahn JG, Marseille E, Auvert B. Cost-effectiveness of male circumcision for HIV prevention in a South African setting. *Public Library of Science Medicine* 2006;3(12):e517.

Kebaabetswe P, Lockman S, Mogwe S, Mandevu R, Thior I, Essex M, Shapiro RL. Male circumcision: an acceptable strategy for HIV prevention in Botswana. *Sexually Transmitted Infections* 2003;79(3):214-9.

Kigozi G, Watya S, Polis CB, et al. The effect of male circumcision on sexual satisfaction and

function, results from a randomized trial of male circumcision for human immunodeficiency virus prevention, Rakai Uganda. *Brit J Urol Int* 2008;101:65-70.

Lagarde E, Dirk T, Puren A, Reathe RT, Bertran A. Acceptability of male circumcision as a tool for preventing HIV infection in a highly infected community in South Africa. *AIDS* 2003;17(1):89-95.

Marck J. Aspects of male circumcision in sub-equatorial African culture history. *Health Transition Review* 1997;7(Suppl):337-60.

Mattson CL, Bailey RC, Muga R, Poulussen R, Onyango T. Acceptability of male circumcision

and predictors of circumcision preference among men and women in Nyanza Province, Kenya. *AIDS Care* 2005;17(2):182-94.

Moses S, Bradley JE, Nagelkerke NJ, Ronald AR, Ndinya-Achola JO, Plummer FA. Geographical patterns of male circumcision practices in Africa: association with HIV seroprevalence. *International* *Journal of Epidemiology* 1990;19(3):693-7.

Ngalande RC, Levy J, Kapondo CP, Bailey RC. Acceptability of male circumcision for prevention of HIV infection in Malawi. *AIDS and Behavior* 2006;10(4):377-85.

Nnko S, Washija R, Urassa M, Boerma JT. Dynamics of male circumcision practices in northwest Tanzania. *Sexually Transmitted Diseases* 2001;28(4):214-8.

Rain-Taljaard RC, Lagarde E, Taljaard DJ, Campbell C, MacPhail C, Williams B, Auvert B. Potential for an intervention based on male circumcision in a South African town with high levels of HIV infection. *AIDS Care* 2003;15(3):315-27.

Scott BE, Weiss HA, Viljoen JI. The acceptability of male circumcision as an HIV intervention

among a rural Zulu population, Kwazulu-Natal, South Africa. *AIDS Care* 2005;17(3):304-13.

UNAIDS. *Collaborating with traditional healers for HIV prevention and care in sub-Saharan Africa: suggestions for program managers and field workers*. Geneva: Joint United Nations Programme on HIV/AIDS; 2006 (Best Practice Collection UNAIDS06.28E / ISBN 92-9-173342-3). (http://data.unaids.org/Publications/IRC-pub07/JC967-TradHealers_en.pdf, accessed 27 February 2007).

van Dam J, Anastasi M-C. *Male circumcision and HIV prevention: directions for future research.* Washington DC: Population Council; 2000 (Horizons Report). (http://www.popcouncil.org/pdfs/circumcision.pdf, accessed 27 February 2007).

Weis HA, Halperin D, Bailey RC, Hayes RJ, Schmid G, Hankins CA. Male circumcision for HIV prevention: From evidence to action? *AIDS* 2008;22:567-78.

Westercamp N, Bailey RC. Acceptability of male circumcision for prevention of HIV/AIDS in sub-Saharan Africa: A Review. *AIDS and Behavior* 2007;11(3):341-55.

Williams BG, Lloyd-Smith JO, Gouws E, Hankins C, Getz WM, Hargrove J, de Zoysa I, Dye C, Auvert B. The potential impact of male circumcision on HIV in sub-Saharan Africa. Public Library of Science Medicine 2006;3(7):e262.

3. APPENDIX B - Male circumcision for HIV prevention publications in chronological order for the past 3 years

[Country experiences in the scale-up of male circumcision in the Eastern and Southern Africa region: two years and counting](http://www.who.int/entity/hiv/pub/malecircumcision/meetingreport_june09/en/index.html)
Meeting report from a sub-regional consultation Windhoek, Namibia 9-10 June 2009
23 July 2009

[Male circumcision situation analysis toolkit](http://www.who.int/entity/hiv/pub/malecircumcision/sit_analysis/en/index.html)
1 May 2009

[Operational guidance for scaling up male circumcision services for HIV prevention](http://www.who.int/entity/hiv/pub/malecircumcision/op_guidance/en/index.html)
WHO and UNAIDS
16 January 2009

[Male circumcision quality assessment toolkit](http://www.who.int/entity/hiv/pub/malecircumcision/qa_toolkit/en/index.html)
A toolkit to assess the safety and quality of services
14 January 2009

[Male circumcision quality assurance guide](http://www.who.int/entity/hiv/pub/malecircumcision/qa_guide/en/index.html)
A guide to enhancing the safety and quality of services
30 October 2008

[Safe, voluntary, informed male circumcision and comprehensive HIV prevention programming](http://www.who.int/entity/hiv/pub/malecircumcision/guide_decision/en/index.html)
Guidance for decision-makers on human rights, ethical and legal considerations
16 September 2008

[Consultation on male circumcision and HIV prevention in the African Region](http://www.who.int/entity/hiv/pub/malecircumcision/meetingreport_apr08/en/index.html)
Meeting report, Brazzaville, Congo, 2-4 April 2008
2 April 2008

[Male circumcision and HIV prevention in Eastern and Southern Africa : communications guidance](http://www.who.int/entity/hiv/pub/malecircumcision/comm_guide/en/index.html)
16 March 2008

[Manual for male circumcision under local anaesthesia](http://www.who.int/entity/hiv/pub/malecircumcision/local_anaesthesia/en/index.html)
WHO | UNAIDS | JHPIEGO | Version 2.5C
29 January 2008

[Male circumcision information package](http://www.who.int/entity/hiv/pub/malecircumcision/infopack/en/index.html)
15 December 2007

[Male circumcision: global trends and determinants of prevalence, safety and acceptability](http://www.who.int/entity/hiv/pub/malecircumcision/globaltrends/en/index.html)
14 December 2007

[Male circumcision quality assurance guidance expert review meeting](http://www.who.int/entity/hiv/pub/malecircumcision/meetingreport_nov07/en/index.html)
Meeting report, Montreux, Switzerland, 12-13 November 2007
12 November 2007

[East and Southern Africa faith-based organizations: Male circumcision consultation](http://www.who.int/entity/hiv/pub/malecircumcision/meetingreport_sep07/en/index.html)
Meeting report, Limuru, Kenya, 20-21 September 2007
20 September 2007

[Male circumcision and HIV prevention: operations research implications](http://www.who.int/entity/hiv/pub/malecircumcision/meetingreport_jun07/en/index.html)
International consultation report | Nairobi, Kenya, 21- 22 June 2007
22 June 2007

[Countries in the Eastern and Southern Africa region agree to accelerate scale up of male circumcision services in the context of HIV prevention](http://www.who.int/entity/hiv/pub/malecircumcision/meetingreport_7may07/en/index.html)
Meeting report, Harare, 7-9 may, 2007
7 May 2007

[New data on male circumcision and HIV prevention:
Policy and programme implications](http://www.who.int/entity/hiv/pub/malecircumcision/research_implications/en/index.html)
6 March 2007

[Social science perspectives on male circumcision for HIV prevention](http://www.who.int/entity/hiv/pub/malecircumcision/meetingreport_jan07/en/index.html)
Meeting report, Durban, 18-19 January, 2007
18 January 2007

[Strategies and approaches for male circumcision programming](http://www.who.int/entity/hiv/pub/malecircumcision/meetingreport_dec06/en/index.html)
Meeting Report, 5 - 6 December 2006, Geneva
6 December 2006

[Regional consultation on safe male circumcision and HIV prevention](http://www.who.int/entity/hiv/pub/malecircumcision/meetingreport_nov06/en/index.html)
Meeting report, Nairobi, Kenya, 20-21 November, 2006
20 November 2006

[Male circumcision: Africa's unprecedented opportunity](http://www.who.int/entity/hiv/pub/malecircumcision/africa_opportunity/en/index.html)
14 November 2006

[The male circumcision and HIV prevention country consultation meeting](http://www.who.int/entity/hiv/pub/malecircumcision/meetingreport_26sep06/en/index.html)
Meeting report, Swaziland, September 26-27 2006
26 September 2006

[Male circumcision and HIV prevention Tanzania country stakeholder consultation](http://www.who.int/entity/hiv/pub/malecircumcision/meetingreport_14sep06/en/index.html)
Meeting report, Tanzania, 14-15 September 2006
14 September 2006

[Male circumcision consultative meeting](http://www.who.int/entity/hiv/pub/malecircumcision/meetingreport_11sep06/en/index.html)
Meeting report, Lusaka, Zambia, 11-12 September 2006
11 September 2006

[Kenya stakeholder consultation on male circumcision in the context of HIV prevention](http://www.who.int/entity/hiv/pub/malecircumcision/meetingreport_6sep06/en/index.html)
Meeting report, Kenya, 6 September 2006
6 September 2006

[Male circumcision and HIV prevention country consultation meetings](http://www.who.int/entity/hiv/pub/malecircumcision/meetingreport_25jul06/en/index.html)
Meeting report, Lesotho, 25 July 2006

Reference

1. Bitega JP, Ngeruka ML, Hategekimana T, Asiimwe A, and Binagwaho A (2011). *Safety and Efficacy Study of the PrePex System for Male Circumcision*. Paper #1007 presented at the 18th Conference on Retroviruses and Opportunistic Infections, Boston MA.

2. A prospective, randomized, open-label trial comparing the PrePex system to surgical circumcision for rapid scale-up of male circumcision in resource limited settings. Protocol RMC-01, Version 1.2. <http://clinicaltrials.gov/ct2/show/NCT01284088>

1. Complete healing was defined as complete epithelialization and no drainage from the wound. [↑](#footnote-ref-2)
